# Eco-friendly fabrication of Ag/Fe_2_O_3_ and Ag/Co nanocomposites *via Anabasis articulata*: GC-MS, phytochemical analysis, antioxidant, antimicrobial, and insecticidal activities

**DOI:** 10.1039/d5ra06599b

**Published:** 2025-10-20

**Authors:** Khaled M. Elattar, Mohammed S. El Hersh, Giuliano Bonanomi, Ahmed M. Abd-ElGawad, Yasser A. El-Amier, Asma A. Al-Huqail

**Affiliations:** a Unit of Genetic Engineering and Biotechnology, Mansoura University El-Gomhoria St. Mansoura 35516 Egypt khaledelattar2@yahoo.com khaledelattar2@mans.edu.eg; b Microbial Activity Unit, Department of Microbiology, Soils, Water and Environment Research Institute, Agricultural Research Center Giza 12619 Egypt m.elhersh@yahoo.com; c Department of Agricultural Sciences, University of Naples Federico II Via Università 100 80055 Portici Italy giuliano.bonanomi@unina.it; d Chair of Climate Change, Environmental Development and Vegetation Cover, Department of Botany and Microbiology, College of Science, King Saud University Riyadh 11451 Saudi Arabia aalhuqail@ksu.edu.sa aibrahim2@ksu.edu.sa; e Plant Production Department, College of Food & Agriculture Sciences, King Saud University P.O. Box 2460 Riyadh 11451 Saudi Arabia aibrahim2@ksu.edu.sa; f Botany Department, Faculty of Science, Mansoura University Mansoura 35516 Egypt yasran@mans.edu.eg

## Abstract

This research reported a novel and sustainable green synthesis of Ag/Fe_2_O_3_ and Ag/Co NCs using *Anabasis articulata* extract. To the best of our knowledge, this is the first report of using *A. articulata* extract as a reducing and stabilizing dual agent in nanocomposite synthesis, which provides an eco-friendly alternative to traditional chemical or physical methods. This approach prevents the use of toxic reagents, minimizes energy requirements, and leverages the phytochemical-dense matrix of the extract to maximize the bioactivity of the resulting nanocomposites. The volatile components of *A. articulata* extract were analyzed by GC-MS analysis. The nanocomposites were characterized by FTIR, UV-Vis, XRD, SEM, EDX, HR-TEM, and zeta potential analysis. The phytochemical analysis of *A. articulata* extract revealed high amounts of phytocomponents (phenolic: 134.13 ± 1.73 mg GAE per g DS), which significantly decreased during the synthesis of nanocomposites. The antioxidant activity tests revealed potent results with IC_50_ at 0.057 ± 1.23 mg mL^−1^ for plant extract, Ag/Fe_2_O_3_ NC (IC_50_ = 0.09 ± 1.02 mg mL^−1^), and Ag/Co NC (IC_50_ = 0.2 ± 1.02 mg mL^−1^) using DPPH assay, which are in agreement with the results of the FRAP assay. Remarkably, Ag/Fe_2_O_3_ NC showed the most noteworthy antibacterial activity, against all types of pathogenic bacteria (19–24 mm). Both *Aphis craccivora* and *Brevicoryne brassicae* exhibited dose-dependent mortality under all treatments, with Ag/Co NC achieving the lowest LC_50_ and highest toxicity index values, slightly surpassing azadirachtin and markedly outperforming Ag/Fe_2_O_3_ NC and the *A. articulata* extract. These findings demonstrate the promise of *A. articulate*-mediated Ag/Fe_2_O_3_ and Ag/Co NCs as potent and promising green nanomaterials, representing the first use of their dual antimicrobial–insecticidal application and underscoring their potential in sustainable pest management strategies.

## Abstract 2

Green-synthesized Ag/Fe_2_O_3_ and Ag/Co NCs using *Anabasis articulata* extract showed strong antioxidant, antibacterial, and insecticidal activities, highlighting their dual eco-friendly potential for antimicrobial and crop protection applications.

## Graphical abstract

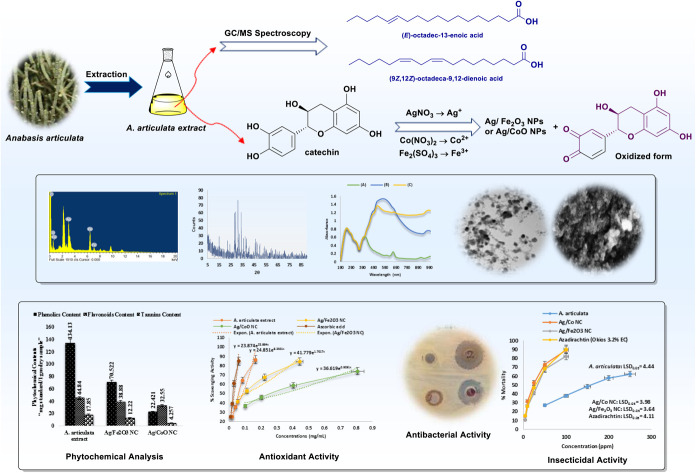

## 1. Introduction

Nanoparticles (NPs) have been established as multi-reckoning materials insofar as they are characterized by specific optical, electrical, magnetic, and chemical traits that render them valuable in other biomedical, pharmaceutical, and ecological processes.^1^ Iron oxide nanoparticles (Fe_2_O_3_) are highly researched owing to their magnetic characteristics, electrical conductivity, and antimicrobial activity, and they are prime candidates for targeted drug delivery, magnetic hyperthermia, and pathogen inhibition.^2^ Likewise, cobalt nanoparticles exhibit superior redox, stability, and catalytic features and outstanding antimicrobial activity with high efficacy against drug-resistant bacteria.^3^ These inbuilt properties of Co nanoparticles and Fe_2_O_3_ position them well to be contenders in the development of multifunctional nanomaterials.

Silver nanoparticles (Ag NPs), on the other hand, have been deeply researched due to their widespread spectrum antimicrobial and antiseptic action and their applications in wound healing, coating, and drugs.^4^ In spite of all the advantages, Ag NPs are confronted with cytotoxicity and even microbial resistance.^5^ Through the combination of Ag with Fe_2_O_3_ or Co in bimetallic nanocomposites, the synergistic antibacterial effect of silver may be utilized, and, at the same time, enhanced stability, magnetic functionality, and biocompatibility are also realized. This new approach offers a new platform for designing nanomaterials with enhanced biological performance over conventional Ag nanoparticles.

Bimetallic nanoparticles (BNPs) are generally more attractive than monometallic nanoparticles (MNPs) because of their enhanced catalytic, optical, and biological activity resulting from the synergistic effect between the two metals.^6^ Their structure and morphology, for instance, core–shell, heterostructured, cluster-in-cluster, or random alloys, are largely regulated by the redox properties of the metals and the type of reducing agents employed.^7^ In green synthesis, plant-derived biomolecules perform the role of reducing and stabilizing agents that promote environmentally benign synthesis of BNPs with enhanced functionality. Among bimetallic nanomaterials, Ag/ZnO NPs have attracted considerable attention owing to their varied applications,^8^ such as analytical sensing, active therapeutics for various diseases, photodynamic therapy,^9^ and photocatalysis.^10^ Furthermore, Ag–Co NC prepared from aqueous extract of *Annona muricata* demonstrated antibacterial activity against *Staphylococcus aureus*, *E. coli*, *Streptococcus pneumoniae*, *Salmonella* sp.,^11^ while Ag–Cu BNPs exhibited broad-spectrum antimicrobial activity through synergistic effects and reactive oxygen species generation.^12^

Medicinal plants are distinguished by having high contents of phytochemicals such as alkaloids, phenolics, flavonoids, triterpenes, saponins, and glycosides, accounting for double roles of acting as reduction and capping agents during BNP synthesis.^13,14^ The genus Anabasis, in particular, is rich in such bioactive metabolites and is therefore a candidate for green synthesis of multifunctional nanomaterials.^15,16^ This is at a time when the immediate challenge of multidrug-resistant (MDR) pathogens limiting the effectiveness of regular antibiotics hangs over the world. Since the new discovery of antibiotics is protracted and costly, nanotechnology-based approaches like plant-mediated synthesis of BNPs arrive as an option to counteract antimicrobial resistance.^17^

Although much has been studied on green-synthesized Ag-based nanoparticles, use of *Anabasis articulata* plant extract for the synthesis of Ag/Fe_2_O_3_ and Ag/Co bimetallic nanocomposites remains unexplored until now. Moreover, less work has drawn a relationship between the contents of phytochemicals in the plant and the structural and functional features of resulting nanomaterials. Ag/Fe_2_O_3_ nanocomposites combine the magnetism of iron oxide with the bactericidal property of silver, with potential applications in targeted delivery and magnetically guided therapy. Similarly, magnetic and oxidative Ag/Co nanocomposites exhibit significant synergistic effects in strong antibacterial activity. Plant-derived phytochemicals, including flavonoids, terpenoids, and phenolic acids, act as natural reducing and capping agents in nanoparticle biosynthesis, controlling particle size, morphology, and stability.

This paper, for the first time, reports the green synthesis of Ag/Fe_2_O_3_ and Ag/Co nanocomposites from *Anabasis articulata* extract by a phytochemical-mediated route. *A. articulata* was selected as it contains a high percentage of phytochemicals, has medicinal significance, and possesses untapped potential as an eco-friendly bioresource for nanomaterial synthesis. The extract was analyzed by GC-MS to identify phytochemical compounds with the potential to act as reducing agents, capping agents, and stabilizers for the nanoparticles. The nanocomposites were exhaustively characterized by utilizing a number of techniques to obtain thorough knowledge regarding their properties. FTIR was employed to identify the functional groups and ensure phytochemical interactions, and UV-Vis spectroscopy was employed to monitor the optical properties and nanoparticle formation. SEM and HR-TEM were employed for the investigation of morphology and particle size, EDX for elemental composition, zeta potential analysis for the assessment of colloidal stability, and XRD for assessing crystallinity. In addition to these descriptions, the biofunctional properties of the nanocomposites were also explored, for example, antioxidant activity using DPPH and FRAP assays, antibacterial activity against pathogen-causing bacteria, and insecticidal activity against two economically important aphid insects, *Brevicoryne brassicae* and *Aphis craccivora*, whose activities are compared with crude extract as well as azadirachtin. To the best of our knowledge, the current is the first-ever report on *A. Articulata*-derived nanocomposites exhibiting dual insecticidal and antibacterial activity, providing mechanistic proof (cuticle disruption, enzyme inhibition, ROS induction) and establishing reference LC_50_, LC_90_, and toxicity index values. Translated to the intersection of phytochemistry and nanotechnology, the present work presents multifunctional nanocomposites as novel answers to the problem of antimicrobial resistance and eco-friendly pest management.

## 2. Materials and methods

### 2.1. Reagents and instruments

Analytical-grade reagents, including Folin-Ciocalteu reagent, gallic acid, DPPH, aluminum chloride, catechin hydrate, vanillin, and ascorbic acid, were sourced from Sigma Aldrich (USA). Sodium carbonate and tannic acid were obtained from El-Nasr Pharmaceutical Chemicals (Egypt), while silver nitrate, cobalt(ii) nitrate, and ferric sulfate were supplied by PIOCHEM (see SI file, Section S1).

GC-MS analysis was performed on a Trace GC-TSQ mass spectrometer (Thermo Scientific, Austin, TX, USA).^18^ UV-Vis spectroscopy was run on Spekol 11, Analytic Jena, Germany to study the optical properties of the nanocomposites. SEM (Czech FEI SEM-type device) provided insights into the morphology of nanoparticles. High-Resolution Transmission Electron Microscope (HR-TEM) was performed on a Thermo Scientific Talos F200i. X-ray diffraction (XRD) experiments were performed on a Pan Analytical Philips. Sonication during synthesis was performed using an Elma Schmidbauer sonicator (Germany), and nanoparticle purification was done *via* centrifugation using a Beckman Coulter Allegra X-15R centrifuge (USA) (see SI file, Section S1).

### 2.2. Extraction of *Anabasis articulata*

*Anabasis articulata* aerial parts were collected from Wadi Hagoul, northern Eastern Desert, Egypt (29°59′46.49′′N 32° 6′0.55′′E), washed, and air-dried. A weighed 10 g of the dried plant materials was mixed with 100 mL ethanol solution (70%).^19,20^ The conical flask containing the mixture was transferred into a horizontal water bath shaker at 220 rpm at 40 °C for 2 h. The mixture was left to cool to room temperature and soaked overnight. The extract was filtered with filter paper and freshly used, and might be stored for more than a week in the refrigerator without losing its efficiency.

### 2.3. Green synthesis of nanocomposites

Silver nitrate solution (100 mL, 10 mM) was prepared in deionized water and stirred at room temperature. *A. articulata* extract (20 mL, 10.58 mg mL^−1^) was added to the stirred silver nitrate solution dropwise, and the mixture was stirred until a brown color was obtained. The mixture was stirred for an additional 2 h until a stable-colored solution was obtained with the formation of Ag NPs. To prepare Ag/Fe_2_O_3_ NC, a solution of ferric sulfate (100 mL, 5 mM) was added to 60 mL of the stirred solution of Ag NPs solution. Ag/Co NC solution was prepared similarly by adding a solution of cobalt nitrate solution (100 mL, 5 mM) into 60 mL of the stirred solution of Ag NPs solution. Both solutions were stirred while rising the temperature to 60 °C for 4 h. The solutions were transferred into a sonicator at 80 °C for 2 h with power. The solid nanomaterials were separated by centrifugation at 10 000 rpm for 10 min, and the residual solids were washed with ethanol, and water to remove contaminated oil, and unreacted materials. The solid nanocomposites were dried at 100 °C in an oven and analyzed for SEM and XRD analyses.^21^

### 2.4. Phytochemical analysis

The tannin content was measured by vanillin–hydrochloride assay.^22^ The reagent was prepared *in situ* with hydrochloric acid at a level of 30% and with 4% of vanillin in methanol. Tannic acid standard curve was plotted to estimate the tannin content (*y* = 0.0009*x*, *R*^2^ = 0.955).^22^ To each of the samples, 100 μL were added through a mixture of a 1 : 10 diluted Folin–Ciocalteu reagent and a sodium carbonate solution. The readings were taken for total phenolic content by incubating the samples at 40 °C for 30 min and measuring absorbance at 765 nm. A standard curve of gallic acid was used to investigate the TPC for each extract (*y* = 0.0062*x*, *R*^2^ = 0.987).^23^ TFC was established by dissolving samples in 100 μL of distilled water with 0.3 mL of 5% sodium nitrite and 0.3 mL of 10% aluminum chloride, after which the mixtures were kept at room temperature for 5 minutes before adding 2 mL of 1 M sodium hydroxide and finally being left for another 10 minutes. When 10 mL had been put into the cuvette, the absorbance was measured at a wavelength of 510 nm. Flavonoid levels were assessed using a catechin standard to obtain the line *y* = 0.0028*x* and the value *R*^2^ = 0.988.^24^ The values were expressed as mg TAE/g DW (TTC), mg GAE/g DW (TPC), and mg CE/g DW (TFC) (for details see SI file, Section S1).

### 2.5. Antioxidant activity

#### 2.5.1. DPPH assay

Antioxidant capacity was analyzed using the DPPH˙ assay, in which the standard ascorbic acid was used.^25^ Serial methanolic dilutions of samples were prepared in methanol and mixed with DPPH˙ solution (0.135 mM). The tubes were incubated for 30 min, and the absorbance was measured at 517 nm. A positive control was prepared using DPPH only in methanol, while methanol was used as a negative control. The % remaining DPPH was calculated as follows (eqn (1)):1
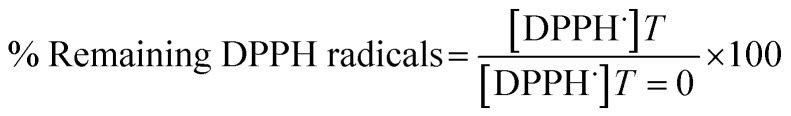
[DPPH˙]*T* is the concentration of DPPH radicals at time *T*. [DPPH˙]*T = 0* is the initial concentration of DPPH radicals (at time 0).

The exponential curve was plotted for sample concentration *versus* the percentage of reaming DPPH for the estimation of IC_50_ values.

#### 2.5.2. Ferric-reducing power assay

The reducing capacity of the extract and nanocomposites was determined by the modified method of a previous study.^26^ The test sample (1 mL) was combined with phosphate buffer (2.5 mL) (0.2 M, pH 6.6) and potassium ferrocyanide (2.5 mL, 1%). The mixture was left for 20 min at 50 °C. Trichloroacetic acid (2.5 mL, 10%) was added to the mixture. Centrifugation at 3000 rpm for 10 min was used to reject the precipitate. Supernatant of 2.5 mL was mixed with 2.5 mL of distilled water. The solution was added with 0.5 mL of 0.1% FeCl_3_, and its absorbance was measured at 700 nm. The measurements of the absorbance were checked with ascorbic acid, a reference standard.

### 2.6. Antibacterial assessment

#### 2.6.1. Agar well diffusion method

To see how the plant extract and nanocomposites affect pathogenic bacteria, the agar well diffusion method was used against eight strains.^27^ Standard antimicrobial susceptibility testing is performed using Mueller–Hinton agar (MHA). This medium was poured into sterilized Petri dishes, allowed it to solidify, and then used for testing. Bacterial cultures were freshly made and diluted to give 1–2 × 10^8^ CFU mL^−1^, which is approximately the 0.5 McFarland standard. Sterile cotton swabs were used to apply each bacterial suspension to the MHA plates, making the bacterial lawn complete all over the plates. Each hole in the agar was punched with an aseptic 9 mm cork borer. One hundred microliters of every test sample at the concentration needed were placed into the wells. The standard antibiotic was tested as a negative control. The plates were placed at 37 °C for an entire day. When the incubation was done, the antibacterial activity was checked by measuring the size of the clear areas surrounding each well. All the experiments were done in sterile environments, and the outcomes were measured in millimeters (mm) to see how good the tested substances were against each of the strains of pathogenic bacteria.

#### 2.6.2. Broth microdilution assay

Minimum inhibitory concentrations (MIC) of Ag/Co and Ag/Fe_2_O_3_ NCs against *K. pneumonia* and *S. aureus* were tested by the broth microdilution method. The nanocomposites were successively diluted two-fold in nutrient broth from 7.09 mg mL^−1^ to 0.0554 mg mL^−1^. A bacterial-grown control nutrient broth without nanocomposites was prepared. Tubes were incubated at 37 °C for 24 h. MIC was established as the lowest concentration of nanocomposite to be visibly seen to inhibit bacterial growth. Turbidity was visually examined before and after incubation, and optical density (OD_600_ nm) was quantified to confirm MIC values.

### 2.7. Insecticidal assay

#### 2.7.1. Rearing of test insects

Colony of *Aphis craccivora* and *Brevicoryne brassicae* was collected from the experimental farm of Mansoura University, Faculty of Agriculture, Egypt. Before experimentation, insect populations were inspected for free insecticide. Cowpea (*Vigna unguiculata*) plants were employed to host *A. craccivora*, whereas cabbage (*Brassica oleracea* var. *capitata*) was employed to host *B. brassicae*. The colonies were kept in controlled laboratory conditions in a plastic house (2.5 × 2.5 × 2.0 m).

#### 2.7.2. Bioassay procedure

Methanolic extract of *Anabasis articulata* was evaluated for insecticidal activity against *A. craccivora* (on cowpea leaves) and *B. brassicae* (on cabbage leaves) by the leaf-spray method. Five concentrations of each plant extract (50–250 ppm) and nanocomposite formulations (5–100 ppm) were prepared in a solution of distilled water containing 0.1% Tween-80 as a surfactant. For each concentration, thirty aphids were used on cowpea or cabbage leaf discs (5 cm diameter) in Petri dishes containing 1.5% agar. Treatment consisted of the spraying of 2 mL of each test solution on aphids with three repetitions per concentration. Control groups were sprayed with distilled water and Tween-80 alone. Treated insects were kept at room temperature, and after 24 h, mortality was counted.

#### 2.7.3. Data analysis

Observed mortality was corrected with Abbott's formula (eqn (2)):^28^2
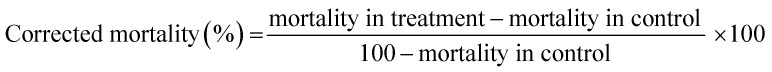
where mortality in treatment = observed mortality (%) in the group exposed to the nanocomposite or extract. Mortality in control = natural mortality (%) observed in the untreated control group.

Concentration-mortality responses were analyzed according to Finney's probit analysis technique^29^ for the calculation of LC_50_ and LC_90_ values at 95% confidence limits and slopes of regression lines (LC-P lines). The toxicity index was calculated based on Sun's equation (eqn (3)):^30^3
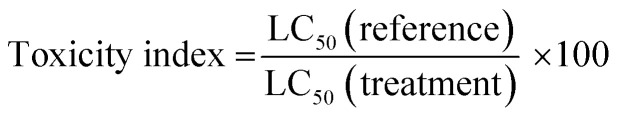
where LC_50_ (reference) = median lethal concentration (ppm) of the reference standard (azadirachtin). LC_50_ (treatment) = median lethal concentration (ppm) of the tested nanocomposite or extract.

### 2.8. Statistical analysis

The results were presented as the average of three replicates. The results were articulated as means ± standard deviation (SD). The mean data of different samples were analyzed by the Statistical Package for Social Sciences (SPSS, version 21). Differences were deliberated statistically significant when *p*-value ≤ 0.05.

## 3. Results and discussion

### 3.1. GC/MS mass spectroscopy

**Fig. 1:**
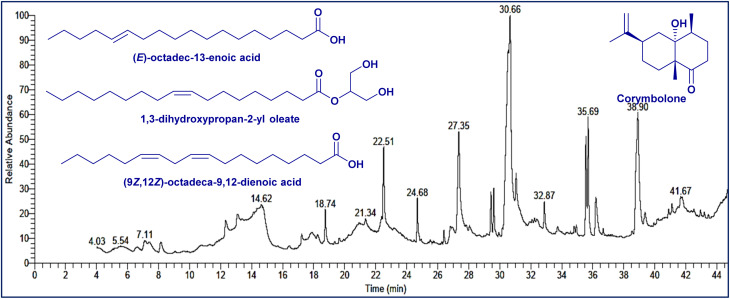
Chromatogram and structures of basic components of the extracted *A. articulata* ethanol extract by GC-MS.

Analysis using GC/MS showed that 28 compounds were found in the ethanolic extract of *Anabasis articulata* which make up 99.96% of the total area percentage (Fig. 1). Six categories of compounds were found: hydrocarbons, terpene, fatty acids, lipids, amino acids, and steroids. Table S1 lists several main compounds together with their retention times, molecular weights, molecular formulae and relative abundance. Many fatty acids and lipids were identified, for example, (*E*)-octadec-13-enoic acid (13.90%), 1,3-dihydroxypropan-2-yl oleate (13.89%) and (9*Z*,12*Z*)-octadeca-9,12-dienoic acid (12.65%). In addition, corymbolone (4.50%) and dehydroxy-isocalamendiol (2.24%) are sesquiterpenes occurring in lower concentrations. Stigmasterol represented steroids, among others, at 1.23%, while ethyl iso-allocholate was at 2.24%. Large amounts of fatty acids (*E*)-octadec-13-enoic acid and (9*Z*,12*Z*)-octadeca-9,12-dienoic acid imply they can be used across various industries because researchers found they have antimicrobial capabilities. The different categories of the compounds are shown in Fig. S1. All the lipids are widely reported for their antioxidant activity, which can be a major contribution to phytochemical-mediated nanoparticle stabilization and reduction. The present analysis summarizes that the ethanol extract of *A. articulata* can be a promising candidate for bioactive molecules for green nanoparticle synthesis with multifaceted applications, including antioxidant, antimicrobial, and insecticidal activities, as demonstrated in the present study.

These are contrary to those reported for stems of *Anabasis articulata*,^31^*Tetraclinis articulata* essential oils, where monoterpenes and sesquiterpenes were predominant,^32^ illustrating the impact of plant species, extraction process (ethanol extract *vs.* essential oil), and environmental factors on chemical composition. Sterol composition here concurs with Ben Menni *et al.*,^33^ where stigmasterol, campesterol, and β-sitosterol were identified as major constituents. Additionally, previous studies have established the antibacterial, antioxidant, and antidiabetic activity of *A. articulata*,^34^ justifying the pharmacological value of the compounds isolated here. In particular, the comparatively high content of fatty acids and the richness of bioactive constituents warrant that ethanol extract of *A. articulata* is a respectable source for the green synthesis and stabilization of nanocomposites.

### 3.2. Mechanism of nanocomposites' formation

**Fig. 2:**
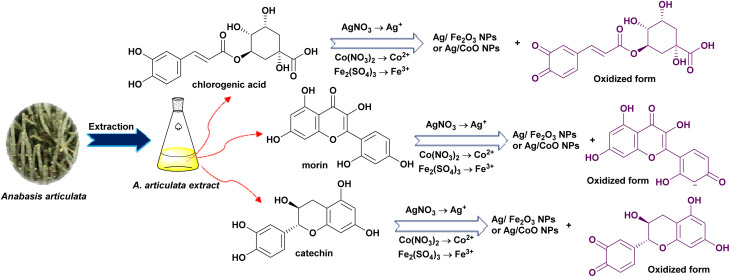
The proposed mechanism for the nanocomposites' formation.

The eco-friendly and sustainable synthesis of novel Ag/Fe_2_O_3_ and Ag/Co NCs was achieved through the interaction of the phytochemical constituents of *A. articulata* extract with metal ions liberated from the precursor metal salt solutions, such as silver nitrate, cobalt nitrate, and ferric sulfate. Mechanism of such interaction can be postulated as follows: the first step consists of reduction of silver ions (Ag^+^) into metallic silver (Ag^0^), accompanied by ionization of cobalt and iron salts to Co^2+^ and Fe^3+^ ions, respectively. The metal ions were directly bioreduced through the interaction with phenol compounds such as gallic acid, chlorogenic acid, caffeic acid, and ellagic acid or flavonoid compounds (*e.g.*, catechin, quercetin, morin, and rutin)^34^ leading to the formation of metal/metal oxide nanoparticles though electron donation with the transformation of these phyto-molecules into their oxidized forms (Fig. 2). Phytochemicals such as catechin and gallic acid are good reducing agents due to their low redox potentials and multiple hydroxyl groups, enabling them to donate electrons to the metal ions. Gallic acid, for example, is oxidatively decarboxylated to give quinone derivatives that act as capping agents and stabilize the nanoparticles.

The critical role of these phytochemicals in this biotransformation is that these compounds can form stable free radicals stabilized by resonance on the benzene ring,^35^ thus facilitating the electron donation process. The role of tannin compounds in this mechanism is that these compounds participate in the nucleation of metal cores and hence control the nanoparticles' size through the chelation process.^36^ This behavior was supported by the presence of poly hydroxy groups attached to aromatic rings. The other phytochemical compounds also participated in the bioreduction and capping processes such as saponins or alkaloids owing to the incorporation of their glycosidic, and nitrogenous groups, respectively.^37^ Terpenes and fatty acids enable the increased stability of the nanoparticles through improving the dispersity and preventing aggregations.^38^ The formation of a thin layer from phytochemicals on the surface of nanoparticles enables stabilization, preventing aggregation, and endorsing colloidal stability. The synergistic interaction of phyto-molecules provided a successful synthesis of multifunctional Ag/Fe_2_O_3_ and Ag/Co NCs with improved structural integrity and biological function.

### 3.3. Characterization of nanocomposites

#### 3.3.1. FTIR spectroscopy

**Fig. 3:**
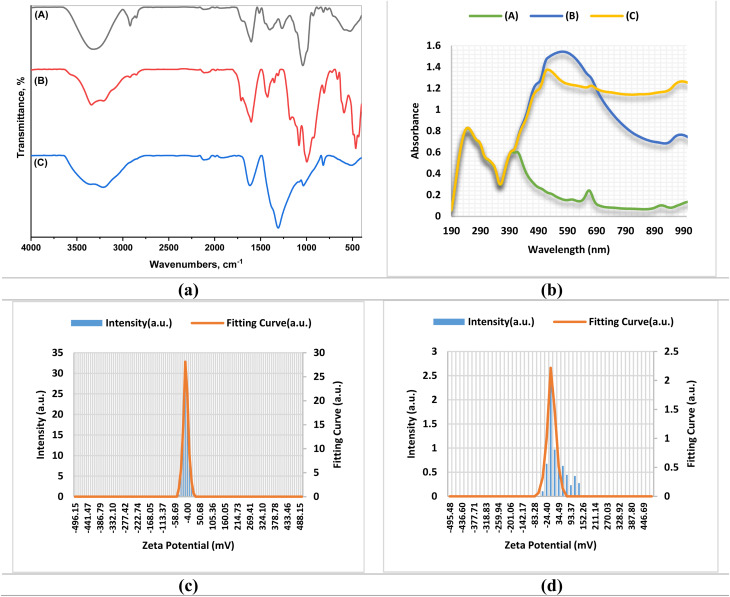
(a) FTIR spectra of the plant extract and nanocomposites. (b) UV-Vis spectroscopy of the investigated samples. (A) Referred to *A. articulata* extract; (B) referred to Ag/Fe_2_O_3_ NC; (C) referred to Ag/Co NC. (c) Zeta potential of Ag/Fe_2_O_3_ NC; (d) zeta potential of Ag/Co NC.

The FTIR spectrum of the plant extract (Fig. 3a and Table S2) showed broad bands at 3346 and 3197 cm^−1^, which are attributed to O–H and N–H stretching vibrations.^39^ The absorption bands at 1715 and 1600 cm^−1^ are due to C=O stretching of carbonyl groups, and aromatic C=C stretching. The absorption bands in the range of 1447–1307 cm^−1^ have been assigned to C–H bending and C–N stretching vibrations. The strong absorption bands at 1183–1024 cm^−1^ are caused by the C–O stretching of alcohols and ethers, whereas absorptions below 900 cm^−1^ have been associated with metal-oxygen and out-of-plane aromatic bending modes.^54^

The FTIR spectrum of Ag/Fe_2_O_3_ NC revealed a similarity in spectral features to the plant extract, proving that phytochemicals of *A. articulata* extract were effectively adsorbed on the nanocomposite surface, leading to stabilization. Peaks in the low-wavenumber region (≤600 cm^−1^) are indicative of Fe–O and Ag–O stretching vibrations, proving the formation of hybrid Ag/Fe_2_O_3_ phases.^40^ For Ag/Co nanocomposite, the broad peak at 3338 cm^−1^ is associated with O–H/N–H stretching vibrations, which indicate remnant plant metabolites on the surface of the nanoparticle. The peak at 1627 cm^−1^ is for C=C and N–H bending, while peaks between 1445-1308 cm^−1^ are for phenolic C–H bending and C–N stretching. The strong band at 1037 cm^−1^ is indicative of the bending C–O vibration, and the 591 cm^−1^ band is specific to Co–O vibration, testifying to cobalt inclusion.^41^ In general, the FTIR findings confirm the observation that bioactive metabolites of *A. articulata* extract are reducing and stabilizing agents in the biosynthesis of Ag/Fe_2_O_3_ and Ag/Co NCs. The functional groups accountable for such observations are in line with earlier findings on plant-mediated synthesis of metal and metal oxide nanoparticles, where phenolics, terpenoids, and proteins are involved in the nucleation and growth stages but inhibit aggregation.^39–41^

#### 3.3.2. UV-vis spectroscopy

UV-Vis spectroscopy was used to examine the optical properties of the *A. articulata* extract and the synthesized Ag/Fe_2_O_3_ and Ag/Co NCs, focusing on surface plasmon resonance (SPR) peaks and absorbance changes that indicate the reduction of metal ions and successful formation of the nanocomposites (Fig. 3b). UV-Visible spectrum of the *A. articulata* extract demonstrated a characteristic absorption peak at 399 nm, relative absorbance of 0.603, indicating the presence of bioactive compounds capable of reducing and stabilizing metal ions during nanocomposite synthesis. Studies have also reported that plant extracts with high levels of phenolic compounds are effective mediators in nanoparticle synthesis.^42^ UV-Vis spectrum of Ag/Fe_2_O_3_ NC revealed a strong peak of absorption at 573 nm with absorbance 1.544, which could be understood by the synergistic effect of Ag and Fe_2_O_3_ components leading to electronic coupling and modification of local dielectric environment. The same phenomena were reported for Ag@α-Fe_2_O_3_ NCs with a shell–core structure, where the SPR band shifts confirmed nanocomposite formation.^43^ The maximum absorption was observed to be at 520 nm at an absorbance value of 1.375 in Ag/Co NC. Redshift is because of electronic coupling between Ag and Co and alteration of the environment dielectric of the nanoparticles. These SPR band shifts are the direct consequences of successful nanocomposite formation, as was previously shown by other authors for metal–metal oxide nanostructures.^44^

#### 3.3.3. Zeta potential analysis

The mean zeta potential was −22.4 mV (mean) with an electrophoretic mobility of −0.000173 cm^2^ V^−1^ s^−1^ for Ag/Fe_2_O_3_ NC, while Ag/Co NC exhibited much lower values of −1.1 mV and −0.000009 cm^2^ V^−1^ s^−1^, respectively (Fig. 3c, d and Table S3). Zeta potential measures nanoparticle surface charge and predicts colloidal stability: above ±30 mV extremely stable, ±10–30 mV fairly stable, and below ±10 mV poorly stable with a tendency to aggregate. Accordingly, Ag/Fe_2_O_3_ NC exhibited medium stability, in accordance with phytochemical capping by *A. articulata* that promoted surface charge formation and dispersion. The same stability was reported for O-carboxymethyl-chitosan-stabilized Ag/Fe_2_O_3_ NC (−25.2 mV),^45^ ginger essential oil (−27.5 mV),^46^ and *Buddleja lindleyana* extract (−24.8 mV).^47^ On the other hand, Ag/Co NC exhibited a quasi-neutral charge, suggesting low electrostatic stabilization and high aggregative tendency. There is little reported literature for Ag/Co NC zeta potential, yet analogous silver-cobalt systems also exhibit low surface charge according to capping agents.^48^ More surface interactions between *A. articulata* phytochemicals and Fe-based oxides are likely reasons for the higher stability of Ag/Fe_2_O_3_ NC compared to Ag/Co NC. Agglomeration of particles can reduce the effective surface area for contact with insect targets and microbial cells and hence inhibit insecticidal and antimicrobial activity. To promote dispersion and bioactivity, strategies like the addition of biocompatible surfactants, polymeric stabilizers, or synthesis condition modification can be employed. These strategies can improve colloidal stability, inhibit agglomeration, and improve the functional performance of Ag/CoO NC.

#### 3.3.4. High-resolution transmission electron microscopy (HR-TEM)

**Fig. 4:**
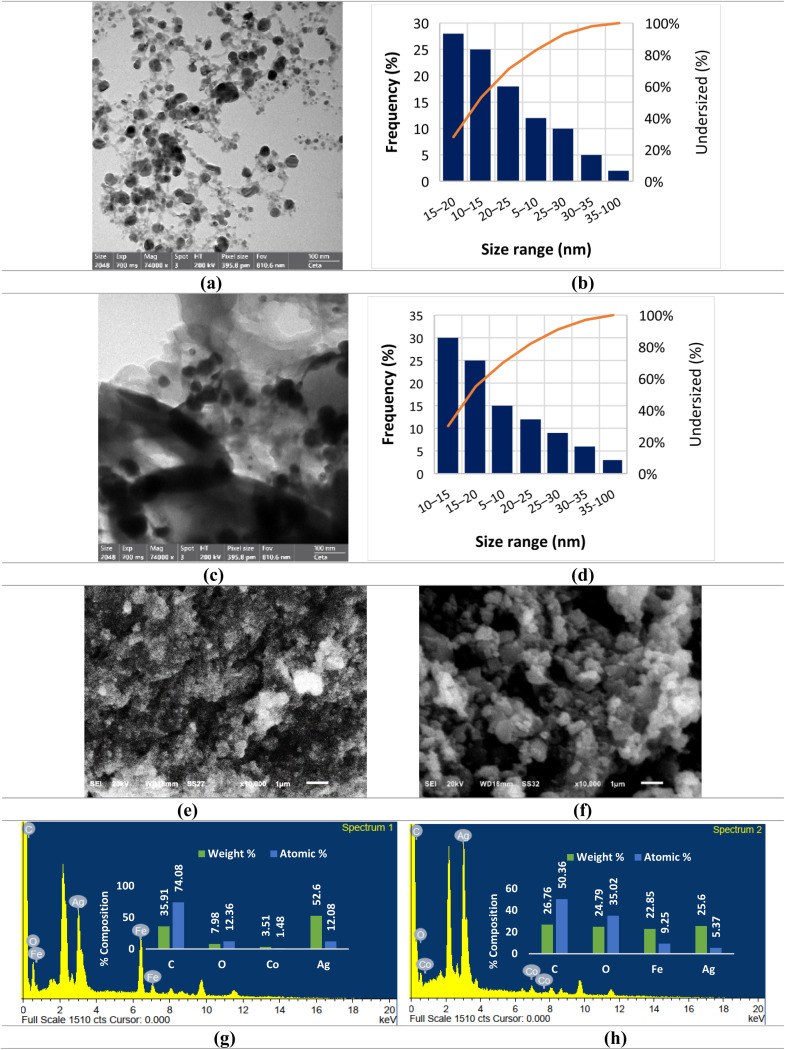
HR-TEM, particle size distribution, SEM and EDX analysis of nanocomposites. HR-TEM micrograph of Ag/Co NC (a). Particle size distribution of Ag/Co NC (b). HR-TEM micrograph of Ag/Fe_2_O_3_ NC (c). Particle size distribution of Ag/Fe_2_O_3_ NC (d). SEM micrograph of Ag/Co NC (e). SEM micrograph of Ag/Fe_2_O_3_ NC (f). EDX analysis of Ag/Co NC (g). EDX analysis of Ag/Fe_2_O_3_ NC (h).

Fig. 4a and S2a show the HR-TEM analysis of Ag/Co NC, where homogeneous dispersions of the nanoparticles are mainly spherical and less than 100 nm in size (Fig. 4b). The micrograph illustrates the homogeneous dispersion of metallic silver (Ag) nanoparticles, as depicted by regions of higher electron density on the cobalt (Co) matrix, which appear as lighter areas because of their lower electron density. This uniform dispersion shows that the Ag and Co components interact well, and there is minimal aggregation. This monodisperse morphology is a result of more efficient coordination of phytochemicals with cobalt ions during synthesis (Section 3.2), allowing controlled nucleation and preventing overgrowth, and therefore resulting in more monodisperse particles.

Fig. 4c and S2b show that, in the case of the Ag/Fe_2_O_3_ NC, there are some nearly spherical and irregular nanoparticles. In most of the areas, the size dispersion of the Ag NPs is more or less homogeneous within the range of 10–20 nm (Fig. 4d), while for Fe_2_O_3_, it is slightly larger, within 20–50 nm. Some areas show minimal agglomeration, especially among the Fe_2_O_3_ particles. Our Ag/Co NCs were relatively more uniformly dispersed and had a narrower particle size distribution than other reported systems, such as Jiang *et al.*^49^ obtained broader sizes and clear clustering in their electrochemically prepared Ag/Co composites. This suggests that the reduction process mediated by phytochemicals applied here promotes more controlled nucleation and stabilization of particles. Similarly, Parveen *et al.*'s^50^ Ag/Fe_2_O_3_ NCs were of irregular shape and underwent extensive agglomeration, consistent with the incomplete surface charge neutralization that we also observed in our system. However, biosynthetic pathway here imparted Ag/Fe_2_O_3_ NCs with relatively more narrow size distributions (10–50 nm) and less extensive agglomeration than,^50^ indicating better phytochemical stabilization. Significantly, these distinctions highlight that green synthesis not only enables effective morphology control of the nanocomposite but also has superiority in synthesis efficiency and stability compared to conventional chemical or electrochemical synthesis.

The higher size distribution is likely to be the result of more efficient adsorption of phytochemicals on Fe–O sites, slowing down particle growth but also neutralizing the surface charge partially, reducing electrostatic stabilization, and leading to agglomeration. Thus, while both nanocomposites were enhanced through phytochemical-mediated reduction and capping, the coordinative chemistry of Co *versus* Fe with polyphenolic groups is the cause of differences seen in observed particle size uniformity and aggregation to a major extent. Generally, HR-TEM analysis confirms the successful synthesis of Ag/Co and Ag/Fe_2_O_3_ nanocomposites whose morphology is directly influenced by phytochemical-metal interaction during synthesis. Briefly, HR-TEM analysis confirmed the successful formation of Ag/Co and Ag/Fe_2_O_3_ nanocomposites with different morphological and structural features.

#### 3.3.5. Scanning electron microscopy (SEM)

The surface appearance and how atoms are grouped in the green-made Ag/Co and Ag/Fe_2_O_3_ nanocomposites were analyzed using SEM analysis (Fig. 4e, f and S3). The scanning was done using 100 00× magnification, 18 mm between the sample and image, and an accelerating voltage of 20 kV. They give useful information about the organization and grouping of the produced substances in material. According to the SEM micrographs of both nanocomposites, the particles are mostly arranged in clusters, having different shapes and sizes. Rather than being well dispersed, the small particles tend to cluster together and unite into bigger aggregates. Most probably, extensive agglomeration happens due to the high surface activity of the nanoparticles, together with a lack of proper stabilization from either steric or electrostatic means during the preparation, regardless of the inclusion of *A. articulata* extract. The shape of the particles ranges from spherical to irregular in the Ag/Fe_2_O_3_ nanocomposites, which is a typical morphology among iron oxide nanomaterials. The clusters create a rough and porous surface, which may lead to a bigger total area and help catalysis or adsorption tasks. Also, these composites show various types and sizes of particles with a high degree of clustering, which indicates they react the same and agglomerate during fabrication.

The SEM analysis of our Ag/Fe_2_O_3_ and Ag/Co nanocomposites revealed aggregated morphologies characterized by irregular particle shapes and broad size distributions. Both materials exhibited significant clustering of nanoparticles, forming larger agglomerates with rough and porous surface textures, typical of biosynthesized nanomaterials due to incomplete stabilization by phytochemicals from *A. articulata*. These findings are consistent with several reports in the literature. For instance, Mosali *et al.*^51^ observed aggregated and irregular particles in Ag/CoFe_2_O_4_/polyaniline composites, even with the presence of a polymeric stabilizer. Similarly, Al-Zahrani *et al.*^46^ and Khan *et al.*^52^ reported agglomerated, quasi-spherical Ag/Fe_2_O_3_ particles synthesized using ginger oil and *Algaia Monozyga* extract, respectively, aligning well with our SEM observations. Otherwise, Guadagnini *et al.*^53^ achieved particles with uniform dispersion and spherical shapes by employing a laser ablation method. The findings show that aggregation appears in most green-synthesized nanocomposites, making them suitable for useful functions in antimicrobial, catalytic, or adsorptive work, despite their structural variations.

Collective characterization findings yielded correlated trends in structural, chemical, and stability analyses. FTIR spectroscopy confirmed the adsorption of *A. articulata* phytochemicals on nanocomposite surfaces as reducing and stabilizing agents during biosynthesis. This behavior was also corroborated through zeta potential measurements, where Ag/Fe_2_O_3_ NC expressed moderate colloidal stability (−22.4 mV), but Ag/CoO NC had quasi-neutral readings (−1.1 mV), reflecting weaker capping and a higher tendency towards aggregation. HR-TEM characterization complemented these findings with properly dispersed spherical Ag/Co NCs (<100 nm) and Ag/Fe_2_O_3_ NCs with bimodal particle sizes (10–20 nm for Ag and 20–50 nm for Fe_2_O_3_) with little or no aggregation. The two nanocomposites were, however, observed to have high agglomeration and irregular morphology under SEM that could be traced back to the failure of sufficient steric or electrostatic stabilization despite phytochemical adsorption. It must be emphasized that precise sizing of agglomerates could not be established from SEM images in themselves since the method only delivers qualitative morphological data, while HR-TEM gives more realistic particle size data. Frequencies of observed agglomeration are greatly influenced by residual phytochemicals of *A. articulata* extract, which are natural capping agents through phenolic, flavonoid, and tannin functional groups. In Ag/Fe_2_O_3_ NC, these phytochemicals are more strongly adsorbed at Fe–O sites, as likewise confirmed by FTIR signals, and partially neutralize surface charges and reduce electrostatic repulsion, leading to larger, denser agglomerates. In Ag/CoO NC, weaker phytochemical adsorption and pseudo-neutral surface charge are to blame for tighter but less stable aggregates. These results validate that the degree and character of residual phytochemical stabilization directly influence nanoparticle dispersion, morphology, and agglomeration and therefore consistently correlate spectroscopic, microscopic, and surface charge results.

##### 3.3.5.1 Energy-dispersive X-ray (EDX)

EDX spectrum was employed to study the elemental composition of the synthesized nanocomposites (Fig. 4g and h). The elemental composition of the Ag/Fe_2_O_3_ NC was mainly carbon (26.76 wt%, 50.36 at%), oxygen (24.79 wt%, 35.02 at%), iron (22.85 wt%, 9.25 at%), and silver (25.61 wt%, 5.37 at%). The high percentages of carbon and oxygen are because the phytochemical residues of *A. articulata* extract, employed as capping and reducing agents during nanoparticle synthesis, contribute to them. The presence of Fe and Ag in considerable ratios confirms the co-formation efficacy of the bimetallic oxide composite. The considerably high percentage of oxygen also confirms partial surface oxidation and the formation of Fe–O and Ag–O bondages, which are in agreement with FTIR results. For Ag/Co NC, EDX spectra showed the following elements: carbon (35.91 wt%, 74.08 at%), oxygen (7.98 wt%, 12.36 at%), cobalt (3.50 wt%, 1.47 at%), and silver (52.60 wt%, 12.08 at%). The much higher carbon content than in Ag/Fe_2_O_3_ NC suggests a denser coating of organic residue from the plant extract that may result in greater particle stabilization and dispersion. Low cobalt content as compared to silver indicates that Ag is the dominant phase of the nanocomposite, and the presence of detectable amounts of oxygen indicates that cobalt oxides are developed on the nanoparticle surface. Concisely, EDX analysis verifies that both nanocomposites consist of respective metallic content and residual bio-organic layers derived from the synthesis medium. Metal and oxygen significantly suggest the presence of metal oxide phases, in line with FTIR and XRD analysis.

#### 3.3.6. X-ray diffraction (XRD)

**Fig. 5:**
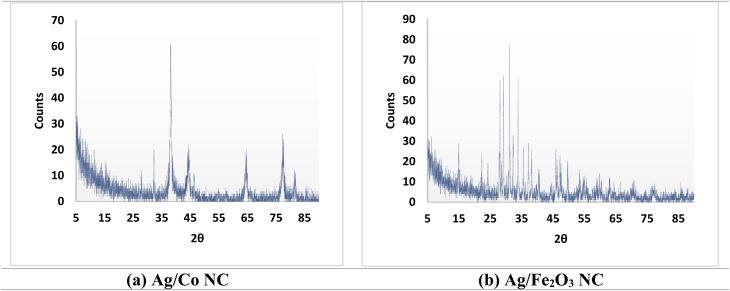
XRD patterns of (a) Ag/Co NC, and (b) Ag/Fe_2_O_3_ NC.

The XRD analysis Ag/Co (Fig. 5a and Table S4) verified clear peaks, in which the results indicate the presence of various crystallographic planes. The maximum 2*θ* (38.1470°) indicates a height of 51.23 by this peak, as well as a Full Width at Half Maximum-FWHM-value of 0.3936°, related to the plane (111) of cobalt (Co). At 32.2830°, 44.3937° and 77.6346°, equally strong peaks at 33.89%, 23.06% and 33.22% intensity, respectively, match the (200), (220) and (311) planes of Co which shows in the figure. The other diffraction maxima were found at 27.8987°, 46.3297° and 64.5517° with relative intensity values of 10.36%, 14.92% and 28.25% for the (101), (102) and (110) planes of Co. Therefore, most of the crystalline Co within the nanocomposite is seen to exist on the plane (111). The pattern of XRD contained peaks at 38.1470° (100% relative intensity), 44.3937° (23.06% relative intensity) and 77.6346° (33.22% relative intensity). So, it can be said that there are silver nanoparticles integrated with the Co matrix. Only the peaks of silver in graphite were present which means the silver remains dispersed and does not develop into large groups. Thus, the typical Scherrer equation was used to estimate the average size of the crystallites in the nanoparticles. In Co, an estimate of average crystallite size of *ca.* 11 nm for the peak at 2*θ* = 44.6° was estimated using the equation mentioned above, assuming JCPDS No. 15-0806. From these peaks at 2*θ* = 38.7°, 44.1°, 64.6°, and 78.3°, the average crystallite size for silver was estimated to be *ca.* 27 nm by using JCPDS No. 03-0931. These values agree with the reported data in the literature for silver nanoparticles.^54^ In conclusion, the performed XRD analysis confirms that crystalline Co with silver nanoparticles in the structure forms the Ag/Co nanocomposite. Well-defined peaks and calculated crystallite sizes point out that the nanocomposite possesses a well-ordered crystalline structure, which is highly important for its prospective applications in different fields.

On the other side, XRD analysis of Ag/Fe_2_O_3_ NC (Fig. 5b and Table S5) revealed multiple peaks showing different diffraction angles where each peak identifies particular crystallographic planes. At a 2*θ* angle of 31.1366° the strongest peak with complete relative intensity of 100% showed a height of 61.46 counts and an FWHM of 0.1181° indicating the (104) plane of iron oxide (Fe_2_O_3_). The X-ray diffraction pattern revealed three key peaks at 33.8900° (89.22%), 28.1323° (73.14%), and 29.2071° (77.71%) for Fe_2_O_3_ with the planes matching (110), (012) and (104). The other Fe_2_O_3_ peaks of high intensity occurred at 32.2928° (39.92%) representing the (113) plane while the peaks at 24.2788° (17.88%) corresponded to the (024) plane and the peak at 22.3139° (31.10%) corresponded to the (104) plane. Three separate XRD pattern peaks were observed at 38.1863° and 23.84% with (111) plane characteristics as well as 44.4047° and 8.10% intensity for the (200) plane while 64.9223° with 11.86% displayed (220) plane features of Ag. The results verified that silver nanoparticles are indeed present in the Fe_2_O_3_ matrix. No other peaks for silver are observed, indicating well-dispersed Ag nanoparticles in the Fe_2_O_3_ matrix, not developing crystalline domains. Following the Scherrer relation between FWHM of diffraction peaks and crystallite sizes, the estimations of Fe_2_O_3_ and silver nanoparticle crystallite sizes were assessed. In this case, a peak at 2*θ* = 35.78° based on JCPDS No. 39-1346 of Fe_2_O_3_ estimated *ca.* 82 nm average size for the Fe_2_O_3_ crystallites. In the case of silver, the average crystallite size was estimated to be *ca.* 27 nm using the peaks at 2*θ* = 38.7°, 44.1°, 64.6°, and 78.3° (JCPDS No. 03-0931). These values fall within the range reported in the literature for silver nanoparticles.^47^ In summary, the XRD analysis confirms the successful synthesis of the Ag/Fe_2_O_3_ nanocomposite, with crystalline Fe_2_O_3_ and silver nanoparticles present in the structure. Well-defined peaks indicate crystallite size in the nanoscale dimension; therefore, good crystalline structure is an index of crystalline ordering for each nanocomposite, representing potential applicative perspectives.

### 3.4. Phytochemical profile

**Fig. 6:**
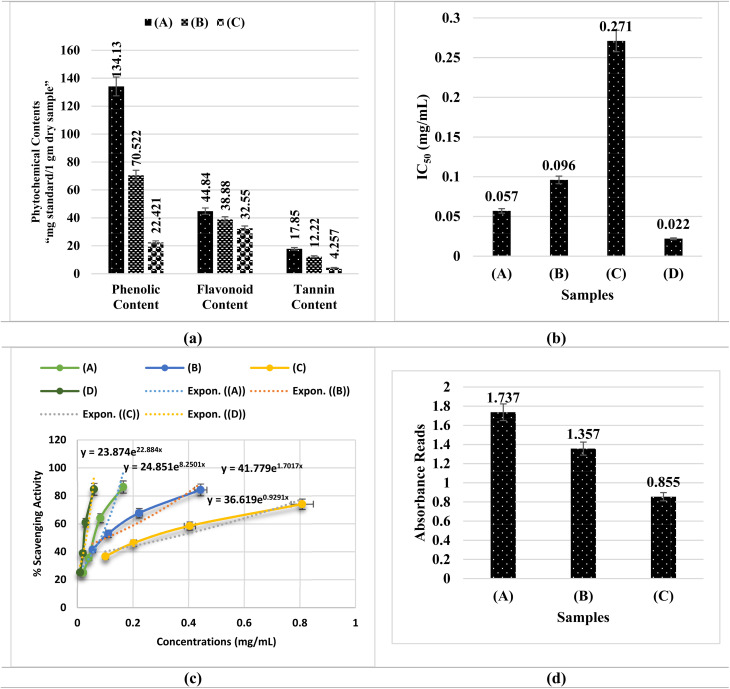
Phytochemical analysis and antioxidant results. (a) A comparison of the phytochemical contents of *A. articulata* extract and the nanocomposites. (b) A comparison between the IC_50_ (mg mL^−1^) values relative to ascorbic acid by DPPH assay. (c) A graph plotted the sample concentration (mg mL^−1^) *versus* % scavenging activity. (d) The antioxidant activity results by ferric reducing power assay. (A) Refers to *A. articulata* extract; (B) refers to Ag/Fe_2_O_3_ NC; (C) refers to Ag/Co NC; and (D) refers to ascorbic acid.

Fig. 6a and Table S6 demonstrate the total amounts of phenolic, flavonoid, and tannin in *A. articulata* extract and its related Ag/Fe_2_O_3_ and Ag/Co NCs. The greatest levels of bioactive components were recorded in the extract: phenols of 134.13 ± 1.73 mg gallic acid per g, flavonoids of 44.84 ± 1.09 mg catechin per g, and tannins of 17.85 ± 0.09 mg tannic acid per g. The highest values ensured that *A. articulata* was rich in phytoconstituents, presumably due to the protective effects of the nanocomposites during their manufacture. The results are in agreement with previous studies. Benhammou *et al.*^55^ and Kambouche *et al.*^56^ also indicated similar higher amounts of phenolic and flavonoid contents in fractions of *A. articulata*, while Al-Joufi *et al.*^34^ further underscored the antibacterial and antioxidant prospects of the plant because of its dense phytochemicals. Likewise, Alamri *et al.*^57^ confirmed that *A. articulata* possesses high phenolic-based antioxidant activity, supporting our results.

Nanoparticle production led to a major decrease in phytochemicals. According to the analysis, only some of the phenolics (70.52 ± 1.24 mg), flavonoids (38.88 ± 1.15 mg), and tannins (12.22 ± 0.07 mg) remain in Ag/Fe_2_O_3_ NC. Photochemical contents were found to be the lowest in Ag/Co NC, as it only recorded 22.42 ± 1.16 mg g^−1^ phenolics, 32.55 ± 1.03 mg g^−1^ flavonoids, and 4.26 ± 0.05 mg g^−1^ tannins. It implies that phytochemicals bind more tightly to Fe_2_O_3_, which might be the result of interactions with nanoparticles or the surface chemistry. When Ag/Fe_2_O_3_ and Ag/Co NCs were synthesized using *A. articulata* extract to prepare them, the concentration of phytochemicals declined considerably, particularly in the case of the Ag/Co system. Phenolic content decreased in the case of Ag/Fe_2_O_3_ and Ag/Co NCs. The loss is in line with trends reported in green nanomaterial research, where phytochemicals from plant extracts are used as reducing agents and capping agents and thus depleted or immobilized on nanoparticle surfaces. Phenolics, flavonoids, and tannins in our system not only acted as reducing and capping agents but were also observed to influence the nucleation and growth of nanoparticles and thus their final size and stability. For instance, the higher retention of phenolics in Ag/Fe_2_O_3_ NC (70.52 ± 1.24 mg g^−1^) compared to Ag/CoO NC (22.42 ± 1.16 mg g^−1^) corresponds to smaller HR-TEM particle diameters and better zeta potential stability found for Ag/Fe_2_O_3_ NC. Particularly, our findings agree with previous observations^58,59^ and reveal that diminished phytochemical content following synthesis proves successful biosynthesis, whereby these compounds depleted in nanoparticle formation and are still partially bound on nanocomposite surfaces. Nanocomposite surfaces with residual phytochemicals are discovered to enhance bioactivity by facilitating electron transfer by phenolic quinones to promote ROS formation, stabilizing the release of Ag^+^ by flavonoid catechol chelation, and promoting bacterial membrane adhesion *via* tannin galloyl functionalization. Therefore, their properties in various uses can be influenced by the phytochemicals that make up Ag/Fe_2_O_3_ and Ag/CoO NCs. Residual phytochemicals on the surfaces of the nanocomposites may further enhance antioxidant and antimicrobial activities by electron transfer, ROS scavenging, and metal and silver phases' synergistic interactions. Phytochemicals thus do not only mediate synthesis but are directly involved in the bioactivity of Ag/Fe_2_O_3_ and Ag/CoO NCs.

### 3.5. Antioxidant activity

#### 3.5.1. DPPH assay

Antioxidant potential of *A. articulata* extract and the nanocomposites prepared (Ag/Fe_2_O_3_ and Ag/Co) was studied through the DPPH free radical scavenging assay (Fig. 6b, c, S4 and Table S7). There was an evident increase in antioxidant activity for *A. articulata* depending on the amount of extract applied. In the highest concentration used (0.165 mg mL^−1^), the extract showed 86.38% DPPH scavenging activity and needed only 0.057 mg mL^−1^ to achieve an IC_50_, demonstrating that it is good at neutralizing free radicals. It is active due to the high phenolic, flavonoid, and tannin contents it has, since they work as good antioxidants. The synthesis of nanoparticles did not change the antioxidant properties much, corresponding to the fall in phytochemical content. The Ag/Fe_2_O_3_ NC provided satisfactory antioxidant activity, at an IC_50_ of 0.09 ± 1.02 mg mL^−1^, but the Ag/Co nanocomposite was not as effective, with an IC_50_ of 0.2 ± 1.02 mg mL^−1^. Considering that the nanocomposites had less activity than the crude extract, the results clearly showed that Ag/Fe_2_O_3_ had significant radical scavenging, reaching the same result as the plant extract and ascorbic acid standard (IC_50_ = 0.022 ± 1.40 mg mL^−1^).

The observed antioxidant activity in the nanocomposites is primarily attributable to the adsorbed or surface-bound phytochemicals during synthesis onto the surface of the nanoparticles. The bioactive molecules, although partially consumed in capping and reduction, are still retained on the surface and can participate in free radical reactions. Additionally, the metal oxide phases (Fe_2_O_3_ and CoO) themselves have the ability to contribute towards radical scavenging in part *via* mechanisms of electron transfer or redox cycling. The DPPH free radical scavenging mechanism is the transfer of hydrogen atoms or electrons by phytochemical compounds to decontaminate the stable DPPH free radical (deep violet) to its reduced form (yellow diphenylpicrylhydrazine). The presence of high phenolic and flavonoid content in *A. articulata* extract allows for efficient hydrogen donation, resulting in efficient and rapid scavenging. In nanocomposites, surface-bound phytochemical residue plays similar roles. Moreover, the Fe^3+^/Fe^2+^ and Co^3+^/Co^2+^ redox pairs in the respective metal oxides can also contribute to single-electron transfer reactions involving radical neutralization. Thus, reduction of antioxidant activity in nanocomposites corresponds to the reduction of available free phytochemicals following synthesis but nevertheless shows successful embedment and preservation of biofunctional moieties. This confirms the double role of *A. articulata* extract as a reducing/stabilizing agent and as a biological activity contributor.

The antioxidant results in our study, which indicated a reduction in the radical scavenging activity in Ag/Fe_2_O_3_ and Ag/Co NCs compared to the *A. articulata* extract, are in good agreement with other studies. Elattar *et al.*^60^ and Ghoniem *et al.*^61^ exhibited the same results, wherein nanocomposites synthesized with *Curcuma longa* or turmeric extract, for instance, Ag/CeO_2_ and Ag@SeO_2_, were less in antioxidant activity compared to the corresponding crude plant extract. In the same vein, Alanazi *et al.*^62,63^ observed that green-synthesized Fe_2_O_3_@CeO_2_-pullulan and Ag@CeO_2_ NCs retained antioxidant activity but in lower levels than the native biopolymer or plant material. These trends are attributed to the use of some of the antioxidant phytochemicals, namely phenolics and flavonoids, during the synthesis of the nanoparticles as they serve as both reducing and capping agents. This account confirms our phytochemical investigation, which confirmed a considerable decrease in total phenolic, flavonoid, and tannin contents in Ag/Fe_2_O_3_ and Ag/Co NCs as compared to the *A. articulata* extract. Thus, the poor antioxidant activity of the nanocomposites is in excellent accordance with the loss of bioactive components following synthesis, confirming the mechanism documented across green nanomaterial literature.

#### 3.5.2. Ferric reducing power assay

Antioxidant activity of *A. articulata* extract and the nanocomposites (Ag/Fe_2_O_3_ and Ag/Co) was once more checked by the Ferric Reducing Power (FRAP) assay. It is an assay that estimates the ability of the antioxidants to reduce ferric ion (Fe^3+^) to ferrous ion (Fe^2+^), reflected by the rising absorbance at 700 nm. As observed in Fig. 6d and Table S8, the *A. articulata* extract exhibited the greatest reducing ability with an absorbance of 1.737 ± 1.27 at a concentration of 10.58 mg mL^−1^. However, Ag/Fe_2_O_3_ NC and Ag/Co NC exhibited lower antioxidant activity with 1.357 ± 1.51 and 0.855 ± 1.08, respectively. The decreased antioxidant capacity of the nanocomposites as compared to the plant extract is consistent with data presented by the DPPH radical scavenging assay. Both antioxidant tests establish that the synthesized nanocomposites, although with residual bioactivity, exhibit a considerable diminishment of free radical scavenging and electron-donating capacities compared to the crude extract. Such a diminishment is directly proportional to the diminishment of total phenolic, flavonoid, and tannin content established in the phytochemical investigation, confirming the role of such molecules as key players for antioxidant activity.

The mechanism of antioxidants in the FRAP assay is primarily electron transfer (ET) mediated, wherein antioxidants donate electrons to ferric ions (Fe^3+^) to convert them into ferrous ions (Fe^2+^).^64^ Phenolic and flavonoid compounds are good reducing agents *via* this mechanism since they carry hydroxyl groups that can donate electrons or hydrogen atoms.^65^ The relatively high absorbance value read in *A. articulata* extract is attributed to its rich composition of phenolic and flavonoids. Conversely, during the biosynthesis of Ag/Fe_2_O_3_ and Ag/Co nanocomposites, a majority of such phytochemicals become oxidized or consumed to reduce metal ions and cap nanoparticles, thereby reducing their availability for antioxidant activity.^66^ The result of the FRP assay is therefore not only in agreement with the findings of the DPPH assay but also in support of phytochemical depletion from the compositional analysis. These results collectively confirm that the green synthesis technique alters the antioxidant profile of the plant-based nanocomposites, although retained activity, particularly in Ag/Fe_2_O_3_ NC, continues to suggest potential biological implications.

### 3.6. Antibacterial activity

**Fig. 7:**
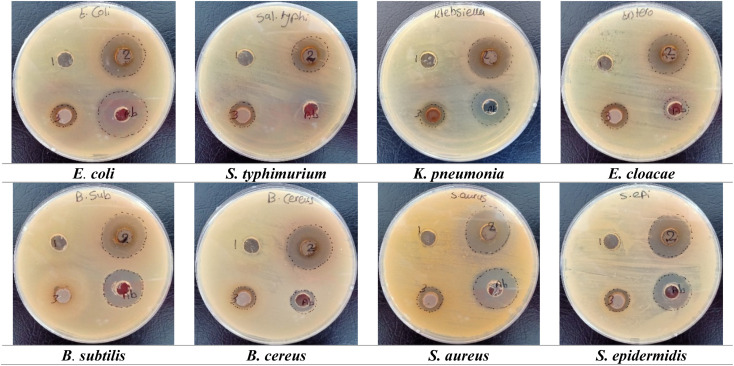
The Petri dish images described the antibacterial activity of the tested samples, and antibiotic against numerous pathogenic bacteria. (1) Referred to *A. articulata* extract. (2) Described to Ag/Fe_2_O_3_ NC. (3) Described to Ag/Co NC, and (Ab) described to antibiotic “azithromycin”.

The antibacterial activity of the *A. articulata* extract and nanocomposites was assessed against different types of pathogenic bacteria by agar well-diffusion assay (Table S9 and Fig. 7, S5). Ag/Fe_2_O_3_ NC revealed excellent antibacterial activity, being more active against Gram-positive bacteria than against Gram-negative bacteria. The presence of Ag and Fe_2_O_3_ nanoferrite in the material suppressed the growth of *B. subtilis*, *B. cereus*, *S. aureus*, and *S. epidermidis*, each with inhibition zone sizes of 24.0 ± 1.83, 24.0 ± 1.61, 24.0 ± 1.72, and 24.0 ± 1.40 mm, respectively. Research indicates Ag/Fe_2_O_3_ NC reduced effectively, when inhibiting *S. typhimurium* growth through antimicrobial action with 19.0 ± 1.52 mm inhibition zone diameter. On the other side, the antibacterial action of Ag/Co NPs proved weaker than the inhibitory behavior of Ag/Fe_2_O_3_ NC. The antibacterial activity of Ag/Co NPs exerted higher effectiveness against *E. cloacae*, resulting in a 15.0 ± 1.64 mm diameter inhibition zone, which matched well with the standard antibiotic azithromycin at 13.0 ± 1.17 mm. The laboratory tests show Ag/Co NPs do not produce noteworthy variations in bacterial inhibition measurements between Gram-positive and Gram-negative strains. The tests using ethanol-extracted *A. articulata* did not show any bacterial growth inhibition for the examined bacterial strains.

Statistical comparison was conducted to identify antibacterial activity of the nanocomposites against the reference antibiotic azithromycin. One-way ANOVA with Tukey's *post hoc* analysis revealed that Ag/Fe_2_O_3_ NC formed significantly greater inhibition zones against Gram-positive microbe (*B. subtilis*, *B. cereus*, *S. aureus*, and *S. epidermidis*) than azithromycin (*p* < 0.05). For Ag/Co NPs, comparable inhibition towards *E. cloacae* (15.0 ± 1.64 mm) to azithromycin (13.0 ± 1.17 mm), and no significant difference (*p* > 0.05) was found. These observations confirm Ag/Fe_2_O_3_ NC to be more active against Gram-positive bacteria, while Ag/Co NPs show moderate activity against chosen Gram-negative bacteria.

The bio-generated silver nanoparticles through green synthesis demonstrated potent antibacterial performance against *E. coli*, *S. aureus*, *K. pneumoniae*, *S. typhi*, and *P. aeruginosa* according to Abdellatif *et al.*,^67^ Mukaratirwa-Muchanyereyi *et al.*,^68^ and Singh & Mijakovic.^69^ The inhibitive mode of Ag NPs against bacterial cells can be related to oxidative stress, protein malfunctioning, membrane distortion, and DNA damage. The biofilm formation of the microbes was inhibited because Ag NPs altered bacterial adhesion.^70^ Moreover, Ag NPs may inhibit respiratory chain dehydrogenase, which interferes with the growth and metabolic process of *S. aureus*. Gram-positive bacteria can also be affected by the mode of action, which causes cellular disruption, depolarization, and instability in *E. coli* and *S. typhimurium*.^71^ Reports show that Ag NPs destroy the bacterial cell membrane and increase the amount of ROS, which leads to their antibacterial properties.^72,73^ The kinetic process of inhibition in *E. coli* may involve the accumulation of Ag NPs in the cell wall and membrane.^74^ In fact, another study by Faisal *et al.*^75^ demonstrated the inhibitory action of iron NPs against *S. aureus* at an inhibition zone of 8 mm. Their study revealed that functionalized iron NPs with aminobenzoic acid enhance the activity of inhibition compared to functionalized iron NPs alone.

It has also been suggested that this process can be due to ROS generation, suppression of enzymes related to respiration, and finally, replication leading to the DNA, causing death. Bimetallic nanoparticles tend to exhibit synergy in an antibacterial effect, while compared to single metal nanoparticles by creating multiple aspects toward bacterial cellular destruction.^76^ In Ag–Au, Ag–Cu, Fe–Ag, and Cu–Ni related studies, these nanoparticles are noted to prevent microbial growth.^77–79^ Likewise, it is emphasized that the use of 50 μg mL^−1^ of CuO–Ag NPs inhibits the growth of *E. coli*, *Salmonella*, and *Listeria*.^80^ When tested at 20 μg mL^−1^, Ag–Pt NPs are more effective in combating the growth of *E. coli* and *S. aureus* species.^81^

In this case, the existence of Ag with Fe_2_O_3_ or Co phases is expected to offer synergistic antibacterial action by enhancing ROS formation, membrane potential damage, and oxidative stress facilitation *via* electron transfer phenomena at the metal–metal oxide interface. Besides, partial leaching of Co^2+^, Fe^3+^, or Ag^+^ ions into the medium is also found to cause microbial inhibition by interacting with thiol-proteins, inactivating key metabolic enzymes, and interfering with nucleic acids. Such synergy indicates that enhanced bioactivity of Ag/Fe_2_O_3_ and Ag/Co nanocomposites is a result of both direct nanoparticle-cell interaction and prolonged ion release, which together provide an antibacterial effect with multi-functionality.

#### 3.6.1. Minimum inhibitory concentration (MIC)

Antibacterial efficiency of prepared nanocomposites was illustrated in their Minimum Inhibitory Concentrations (MICs) against *K. pneumoniae* and *S. aureus* (Tables 1 and S10–S13). Ag/Fe_2_O_3_ exhibited MIC at 1.7725 mg mL^−1^ for both the bacteria, with comparable performance whether Gram-negative or Gram-positive. Ag/Co illustrated far more activity against *K. pneumoniae* with a far lower MIC value of 0.2216 mg mL^−1^, bearing witness to its increased antibacterial activity against Gram-negative bacteria. Its activity against *S. aureus* was weaker and needed a higher concentration of 3.545 mg mL^−1^ to inhibit completely. The findings demonstrate that although both nanocomposites are active, their activities are strain-specific, with Ag/Co being more active and selective against *K. pneumoniae*, and Ag/Fe_2_O_3_ being balanced in activity against the two pathogens. These variations observed are attributed to the variations in bacterial cell wall chemical composition and synergistic action of Ag with Fe_2_O_3_ or Co, which are responsible for ion release, ROS generation, and microbial membrane interactions. This establishes the feasibility of designing nanocomposite materials for the attainment of desired antibacterial activity against a range of pathogens.

**Table 1:** Minimum Inhibitory Concentration (MIC) results of Ag/Fe_2_O_3_ and Ag/Co nanocomposites against *K. pneumoniae* and *S. aureus*

Bacteria	Nanocomposite	MIC (mg mL^−1^)	MIC tube no.	Observation (no turbidity)
*K. pneumoniae*	Ag/Fe_2_O_3_	1.7725	3	Growth inhibited
*K. pneumoniae*	Ag/Co	0.2216	6	Growth inhibited
*S. aureus*	Ag/Fe_2_O_3_	1.7725	3	Growth inhibited
*S. aureus*	Ag/Co	3.545	2	Growth inhibited

### 3.7. Insecticidal activity

**Fig. 8:**
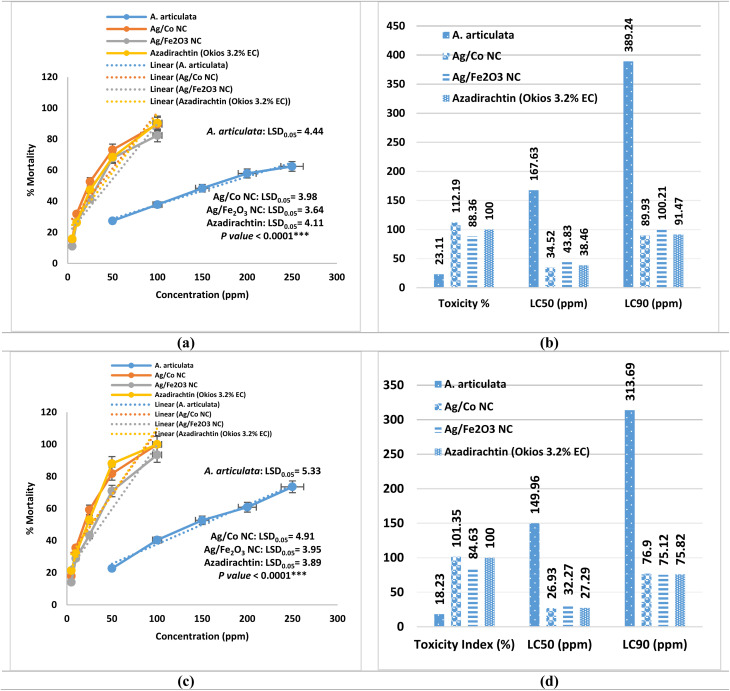
Toxicity on *Aphis craccivora* and *Brevicoryne brassicae* after one day under laboratory conditions. (a) Dose-response toxicity of *A. articulata* extract and nanocomposites on *Aphis craccivora* after 24 h, with fitted curves and shaded areas representing the 95% confidence intervals. (b) A comparison of the toxicity index %, LC_50_ (ppm) and LC_90_ (ppm) upon treatment against *Aphis craccivora*. (c) Dose-response toxicity of *A. articulata* extract and nanocomposites on *Brevicoryne brassicae* after 24 h, with fitted curves and shaded areas representing the 95% confidence intervals. (d) A comparison of the toxicity %, LC_50_ (ppm), and LC_90_ (ppm) upon treatment against *Brevicoryne brassicae*. % Mortality was expressed as mean ± SE (standard error) of 3 replicates. LC_50_; LC_90_; upper limit; lower limit; their confidence limits at 95%. Different letters per each treatment (Tables S14 and S15) mean values significance at a probability level of 0.05. ***p < 0.0001.

Insecticidal activity of *A. articulata* extract, Ag/Co NC, Ag/Fe_2_O_3_ NC, and azadirachtin (Okios 3.2% EC) against *Aphis craccivora* was evaluated in the laboratory after 24 h exposure (Table S14 and Fig. 8a, b). Mortality caused by all the treatments was concentration-dependent, which increased with increase in dose (*p* < 0.0001). Among the tested compounds, Ag/Co NC was most potent with LC_50_ and LC_90_ values of 34.52 and 89.93 ppm, respectively, and a toxicity index of 112.19%. These were lower than those for azadirachtin (LC_50_ = 38.46 ppm; LC_90_ = 91.47 ppm; toxicity index = 100%), which testified to the high efficacy of Ag/Co NC. The high slope (3.04 ± 0.40) of the probit regression informs us of the sharp dose-mortality reaction and high reliability of the estimates of lethality. Third in toxicity was Ag/Fe_2_O_3_ NC with LC_50_ = 43.83 ppm; LC_90_ = 100.21 ppm; toxicity index = 88.36%, with greater insecticidal activity than crude *A. articulata* extract but reduced potency compared to Ag/Co NC and azadirachtin. Its regression slope (2.83 ± 0.35) and high correlation coefficient (*R* = 0.9413) denote a stable concentration-mortality relationship. The crude *A. articulata* extract, on the other hand, exhibited the least effect, LC_50_ = 167.63 ppm and LC_90_ = 389.24 ppm, and thus a toxicity index of only 23.11%. Although much less effective, the performance aligns with previous studies on the antagonistic aphicidal activities of plant extracts.^82,83^ Order of efficacy, therefore LC_50_ values, therefore was: Ag/Co NC > azadirachtin > Ag/Fe_2_O_3_ NC > *A. articulata* extract.

At highest concentrations tested, Ag/Co NC induced 89.62 ± 2.88% mortality that was statistically equivalent to azadirachtin (90.37 ± 2.05%) but significantly higher than Ag/Fe_2_O_3_ NC (82.42 ± 2.34%) and the plant extract (62.47 ± 1.98%). These results corroborate earlier findings that nanocomposites tend to surpass conventional botanicals in pest control due to their enhanced surface reactivity, penetration of cuticular barriers, and long-release behavior potential.^84^ Furthermore, the higher activity of Ag/Co NC compared to Ag/Fe_2_O_3_ NC demonstrates a synergistic effect of cobalt on enhancing insecticidal efficacy.

These findings agree with previous statements that metal nanoparticles and nanocomposites increase insecticidal activity much more than phytochemical formulations but still conform to the philosophy of green pest management.^85,86^ Interestingly, although azadirachtin remains an intense standard due to the extensive documentation of its antifeedant and growth-disrupting effects,^82^ the comparably high efficacy of Ag/Co NC at lower application rates indicates the high worth of this nanoinsecticide as a sustainable agent.

On the other hand, the toxicity tests revealed that all the agents tested, *A. articulata* extract, Ag/Co NC, Ag/Fe_2_O_3_ NC, and azadirachtin, induced severe, concentration-dependent mortality of *B. brassicae* within 24 h (*p* < 0.0001; Table S15 and Fig. 8c). The most effective was Ag/Co NC with an LC_50_ of 26.93 ppm and an LC_90_ of 76.90 ppm, slightly greater than azadirachtin (LC_50_ = 27.29 ppm; LC_90_ = 75.82 ppm) (Fig. 8d). Both the treatments were 100% mortal at 100 ppm, although Ag/Co NC was slightly more active at sub-optimal concentrations, supported by its highest toxicity index (101.35%). Ag/Fe_2_O_3_ NC ranked third (LC_50_ = 32.27 ppm; LC_90_ = 75.12 ppm; toxicity index = 84.63%), similar to that of Ag/Co NC and azadirachtin at high concentrations, but less active at moderate levels. The crude A. *A. articulata* extract was less effective (LC_50_ = 149.96 ppm; LC_90_ = 313.69 ppm; toxicity index = 18.23%), with only 73.54% mortality at 250 ppm. This finding is in agreement with earlier work that crude plant materials require higher doses or extended exposure time for effective aphid suppression.^82,87^ In order of efficacy in decreasing potency by LC_50_ values was: Ag/Co NC > azadirachtin > Ag/Fe_2_O_3_ NC > *A. articulata* extract.

The amplified activity of Ag/Co NC reflects the synergistic interaction between silver and cobalt nanoparticles that has been reported to have improved insecticidal effects by multiple mechanisms like cuticular disruption, the induction of oxidative stress, and the disruption of detoxifying enzymes.^84–86^ Equivalent Ag/Fe_2_O_3_ NC high lethality shows the general promise of metal nanocomposites, even though slight differences in surface chemistry and ROS generation can explain its lower efficacy relative to Ag/Co NC.^88^ The high efficacy of azadirachtin is consistent with its reported antifeedant and hormonal interference activity.^89^ Particularly, these results justify that nanocomposite formulations, particularly Ag/Co NC, are as effective or better than commercial botanical insecticides in rapid aphid control and can result in the utilization of lower dosages and environmental load in integrated pest management programs.

The higher activity of Ag/Co NC may be due to various mechanisms: (i) generation of ROS, inducing oxidative stress and apoptosis; (ii) disruption of the cuticle, allowing the entry of ions; (iii) inhibition of detoxifying enzymes; and (iv) interference with nutrient uptake and neural transmission.^84,86^ Cobalt was observed to enhance the generation of ROS, which is responsible for its higher activity compared to Ag/Fe_2_O_3_ NC. For comparison, azadirachtin has a mainly antifeedant and growth regulator function, interfering with hormonal and molting processes,^82^ while crude plant extracts rely on intricate phytochemicals with minimal direct toxicity. The quality and rapidity of Ag/Co NC action underline its promise as a green nanoinsecticide; however, further verification *via* field trials is necessary to ensure environmental safety.

## 4. Conclusion

This study showed that *Anabasis articulata* extract can be used as an eco-friendly way to create Ag/Fe_2_O_3_ and Ag/Co NCs. The detailed characterization established that crystalline nanoparticles of varied morphology and stabilities were formed. HR-TEM indicated highly dispersed spherical Ag/Co NCs (<100 nm) and Ag/Fe_2_O_3_ NCs with Ag (10–20 nm) and Fe_2_O_3_ (20–50 nm) domains with moderate aggregation, confirming nanocomposite formation but suggesting the employment of stabilizers for enhancement in dispersion. Zeta potential measurement (moderately stable Ag/Fe_2_O_3_ NCs: −22.4 mV *vs.* low stability of Ag/Co NCs: −1.1 mV) and XRD analysis detected highly ordered crystalline structures with average crystallite dimensions of ∼11 nm (Co) and ∼27 nm (Ag) in Ag/Co NC, and ∼82 nm (Fe_2_O_3_) and ∼27 nm (Ag) in Ag/Fe_2_O_3_ NC, reflecting their nanoscale structural integration and possible bioactivity. Phytochemical analysis showed the plant extract to be rich in phenolics, flavonoids, and tannins, which are all of interest concerning reduction and capping reactions during the biosynthetic process. Quantitative determinations indicated the extract to be 134.13 ± 1.73 mg GAE per g DW phenolics, 44.84 ± 1.09 mg CE per g DW flavonoids, and 17.85 ± 0.09 mg TAE per g DW tannins. The new nanocomposites, notably Ag/Fe_2_O_3_ NC, still had impressive antioxidant activities (DPPH assay: IC_50_ = 0.096 ± 1.02 mg mL; FRAP assay: absorbance = 1.357 ± 1.51), despite the initial substances and antioxidant activities of the extract decreasing with synthesis. For example, Ag/Fe_2_O_3_ NC scavenged 84.31 ± 1.33 DPPH radicals at 0.443 mg mL^−1^, which was superior to Ag/CoO NC. It is interesting to note that the fabricated nanocomposites were found to have superb antibacterial activity, and Ag/Fe_2_O_3_ NC was found to inhibit both bacteria most intensely compared to the inhibitory effect of Ag/CoO NC and the plant extract. Specifically, Ag/Fe_2_O_3_ NC was found to have the largest inhibition zones of 22.0 ± 1.38 mm and 24.0 ± 1.72 mm against *E. coli* and *S. aureus*, respectively, in the case of Ag/CoO NC (13.0 ± 16.3 and 14.0 ± 1.07 mm) and the extract (no activity). The enhanced antimicrobial activity is likely a result of synergistic effects between silver nanoparticles, metal oxides, and residual phytochemicals. Amongst the agents considered, Ag/Co NC proved to be the most toxic against *Aphis craccivora* and *Brevicoryne brassicae*, surpassing azadirachtin, Ag/Fe_2_O_3_ NC, and comparatively less active *A. articulata* extract. LC_50_ values for Ag/Co NC were 34.52 mg L^−1^ (*A. craccivora*) and 26.93 mg L^−1^ (*B. brassicae*), whereas these were 43.83 mg L^−1^ and 32.27 mg L^−1^ for Ag/Fe_2_O_3_ NC, and >120 mg L^−1^ for the crude extract. Its improved efficacy may be due to ROS production, disruption of the cuticle, and enzyme inhibition, though these mechanisms are hypothetical. To validate these hypotheses, future studies ought to use biochemical and physiological bioassays (*e.g.*, ROS determination, inhibition of detoxifying enzymes, ultrastructure imaging of the cuticle). Field testing is also highly crucial to identify application rates and ecological safety, thereby validating Ag/Co NC as an eco-friendly nanoinsecticide for pest management. Green-synthesized nanocomposites also hold the promise of broader applications in biomedicine (*e.g.*, targeted drug delivery, biosensing, antibacterial dressings, wound healing formulations, and coating of implants), in addition to their applications in environmental detoxification and antimicrobial therapy. There should be further refinement of synthesis conditions for enhanced stability and dispersibility, development of mechanistic insight into action, and determination of efficacy in *in vivo* models in future studies.

## Conflicts of interest

The authors declare that they have no competing interests.

## Supplementary Material

RA-015-D5RA06599B-s001

## Data Availability

All data supporting the findings of this study are available within the article and its supplementary information (SI) file. Supplementary information: “Section S1: Materials and methods” and “Section S2: Results.” Section S1 covers the details of chemicals and reagents, instruments (models), the procedure of GC-MS analysis, and bacterial species with accession numbers. Section S2 presents detailed results from GC-MS interpreted results (Table S1 and Fig. S1), FTIR analysis (Table S2), zeta potential analysis (Table S3), HR-TEM and SEM micrographs (Fig. S2 and S3), XRD analysis (Tables S4 and S5), phytochemical analysis (Table S6), and antioxidant activity assays (Table S7, Fig. S4, Table S8), along with the antibacterial activity results (Table S9), bacterial species used for testing, as well as a comparison of the antibacterial results (Fig. S5). Tables S10–S13 presented the results of the MIC test. The results of insecticidal activity on *Aphis craccivora* and *Brevicoryne brassicae* are presented in Tables S14 and S15. See DOI: https://doi.org/10.1039/d5ra06599b.
