# Supplementary material for: Eco-friendly fabrication of Ag/Fe2O3 and Ag/Co nanocomposites via Anabasis articulata: GC-MS, phytochemical analysis, antioxidant, antimicrobial, and insecticidal activities
Source: RSC Adv. 2025 Oct 20;15(46):39336–54. doi: 10.1039/d5ra06599b (PMC12536295; doi:10.1039/d5ra06599b)
Supplement: RA-015-D5RA06599B-s001 [file RA-015-D5RA06599B-s001.pdf]

Supplementary Information (SI) for RSC Advances.
This journal is © The Royal Society of Chemistry 2025

# Supplementary Data

# Eco-Friendly Fabrication of Ag/$Fe_2O_3$ and Ag/Co Nanocomposites *via* *Anabasis articulata*: GC-MS, Phytochemical Analysis, Antioxidant, Antimicrobial, and Insecticidal Activities

## Section S1: Materials and Methods

**Chemicals and Reagents**:

Folin-Ciocalteu reagent (analytical grade, Fluka, Biochemical Inc., Bucharest, Romania), Gallic acid (≥98%, Biomedical Inc., Orange City, FL, USA), 1,1-Diphenyl-2-picrylhydrazyl ($DPPH^{\bullet}$) (≥97%), aluminum chloride (anhydrous, ≥99%), sodium hydroxide (pellets, ≥99%), sodium nitrite (≥99%), catechin hydrate (≥98%), vanillin (≥99%), hydrochloric acid (37%), and ascorbic acid (≥99%) were purchased from Sigma Aldrich (St. Louis, USA). Sodium Carbonate (analytical grade), and tannic acid (≥98%) were purchased from El-Nasr Pharmaceutical Chemicals, Cairo, Egypt. Silver nitrate ($AgNO_3$, 99%), cobalt(II) nitrate ($Co(NO_3)_2$, 99%) and Ferric sulfate ($Fe_2(SO_4)_3$, ≥ 98%) were purchased from PIOCHEM laboratory chemicals.

**Instruments**

UV-Vis spectroscopy (Spekol 11, Analytik Jena AG, Jena, Germany) was used to investigate optical properties. At the same time, SEM (Czech FEI SEM-type instrument) provided insights into the morphology of the nanoparticles. To characterize nanoparticles, a high-resolution transmission electron microscope (HR-TEM) was performed on Thermo Scientific Talos F200i. X-ray diffraction (XRD) analyses were executed on a Pan Analytical Philips. Sonication was performed using an Elma Schmidbauer GmbH (Gottlieb-Daimler-Straße 17, Singen, Germany) sonicator during nanoparticle preparation. Subsequently, a Beckman Coulter Allegra X-15R centrifuge (Beckman Coulter, Inc., California, USA) was employed to isolate the purified nanoparticles.

**Gas chromatography-mass spectrometry analysis (GC-MS)**

The chemical composition of the extracted *A. articulata* was described by employing the ethanol extract on a Trace GC-TSQ mass spectrometer (Thermo Scientific, Austin, TX, USA) with a direct capillary column TG-5MS (30 m x 0.25 mm x 0.25 m film thickness) [1]. The temperature of the column oven was first maintained at 50 °C, then raised by 5 °C per minute to reach 250 °C and maintain for 2 minutes and then increased by 30 °C per minute to reach the

final temperature of 300 °C and maintain for 2 minutes. The MS transfer line and injector were kept at temperatures of 260 and 270 °C, respectively. As a carrier inert gas, helium (He) was employed at a constant flow rate of 1 mL/min. After 4 minutes, the solvent was removed, and 1 l diluted samples were immediately fed into the GC in split mode using an Autosampler AS1300. In packed scan mode, EI mass spectroscopy data over the *m/z* range of 50-500 was gathered at an ionization voltage of 70 EV. The ion source's temperature was set at 200 °C. By comparing the mass spectral data of the various extracted plant materials to those of the mass spectrometry databases WILEY 09 and NIST 14, it was possible to understand the chemical composition of each of the distinct plant materials. Five potential components were suggested by the GC-MS analysis for each identified peak. The probability factors and the primary structure's fragmentation patterns governed the chosen structure in the case of the different suggested components.

**Bacterial species**:

Gram-negative bacteria (*E. coli* ATCC 10536, *S. typhimurium* ATCC 25566, *K. pneumoniae* ATCC 10031, and *E. cloacae* DMS 30054) and Gram-positive bacteria (*B. subtilis* DMS 1088, *B. cereus* EMCC 1080, *S. aureus* ATCC 6538, and *S. epidermidis* EMCC 1353t) were obtained from the Microbiological Resources Centre, Cairo, Egypt (Mircen), and the American Type Culture Collection (ATCC, Manassas, VA, USA).

## Section S2: Results

### GC/MS Spectroscopy

**Table S1**. The results of GC/MS spectroscopy of the volatile components identified from of *A. articulata* ethanol extract.

| Entry | Compound name | Classification | Retention time (RT, min) | Molecular Weight | Molecular formula | Area % |
|---|---|---|---|---|---|---|
| **1** | 3,5-Dihydroxy-6-methyl-2,3-dihydro-4*H*-pyran-4-one | Hydrocarbon | 8.13 | 144 | $C_6H_8O_4$ | 0.90 |
| **2** | Cumic alcohol | Monoterpene | 12.31 | 150 | $C_{10}H_{14}O$ | 1.28 |
| **3** | 2-ethoxy-5-methyl-3-methylene-3,4-dihydro-2H-pyran | Hydrocarbon | 13.08 | 154.21 | $C_9H_{14}O_2$ | 1.18 |
| **4** | 1,4-dimethoxybutane-2,3-diol | Hydrocarbon | 14.63 | 150.17 | $C_6H_{14}O_4$ | 2.23 |
| **5** | 6-allyl-4-methoxy-benzo[d][1,3]dioxole | Hydrocarbon | 17.22 | 192.21 | $C_{11}H_{12}O_3$ | 0.73 |
| **6** | Diethyl phthalate | Hydrocarbon | 18.74 | 222.24 | $C_{12}H_{14}O_4$ | 2.02 |
| **7** | Corymbolone | Sesquiterpene | 22.51 | 236.36 | $C_{15}H_{24}O_2$ | 4.50 |
| **8** | diisobutyl phthalate | Hydrocarbon | 24.68 | 278.35 | $C_{16}H_{22}O_4$ | 3.07 |
| **9** | Methyl palmitate | Lipid | 26.40 | 270.46 | $C_{17}H_{34}O_2$ | 0.87 |
| **10** | Dehydroxy-isocalamendiol | Sesquiterpene | 26.81 | 220.36 | $C_{15}H_{24}O$ | 2.24 |
| 11 | Palmitic acid | Fatty acid | 27.36 | 256.43 | $C_{16}H_{32}O_2$ | 6.73 |
| 12 | methyl (7E,10E)-octadeca-7,10-dienoate | Lipid | 29.43 | 294.48 | $C_{19}H_{34}O_2$ | 2.79 |
| 13 | methyl (E)-octadec-13-enoate | Lipid | 29.61 | 296.50 | $C_{19}H_{36}O_2$ | 2.61 |
| 14 | (9Z,12Z)-octadeca-9,12-dienoic acid | Fatty acid | 30.54 | 280.45 | $C_{18}H_{32}O_2$ | 12.65 |
| **15** | (E)-octadec-13-enoic acid | Fatty acid | 30.67 | 282.47 | $C_{18}H_{34}O_2$ | 13.90 |
| **16** | Oleic acid | Fatty acid | 31.05 | 282.47 | $C_{18}H_{34}O_2$ | 2.22 |
| **17** | Glycidyl palmitate | Lipid | 32.87 | 312.49 | $C_{19}H_{36}O_3$ | 1.74 |
| **18** | (Z)-7-methyltetradec-8-en-1-yl acetate | Lipid | 33.73 | 268.44 | $C_{17}H_{32}O_2$ | 0.63 |
| **19** | ethyl (9Z,12Z)-octadeca-9,12-dienoate "Ethyl linoleate" | Lipid | 34.80 | 308.51 | $C_{20}H_{36}O_2$ | 0.51 |
| **20** | 1,3-dihydroxypropan-2-yl (9Z,12Z)-octadeca-9,12-dienoate | Lipid | 35.56 | 354.53 | $C_{21}H_{38}O_4$ | 6.36 |
| **21** | Glycidyl oleate | Lipid | 35.69 | 338.53 | $C_{21}H_{38}O_3$ | 7.42 |
| **22** | 2,3-dihydroxypropyl palmitate | Lipid | 36.18 | 330.51 | $C_{19}H_{38}O_4$ | 3.06 |
| **23** | O-benzyl-L-serine | Amino acid | 36.67 | 195.22 | $C_{10}H_{13}NO_3$ | 0.42 |
| **24** | 1,3-dihydroxypropan-2-yl oleate | Lipid | 38.90 | 356.55 | $C_{21}H_{40}O_4$ | 13.89 |
| **25** | Ethyl iso-allocholate | Steroid | 39.36 | 436.63 | $C_{26}H_{44}O_5$ | 2.24 |
| **26** | 2,3-bis[(trimethylsilyl)oxy]propyl (9E,12E,15E)-9,12,15-octadecatrienoate | Lipid | 40.88 | 496.0 | $C_{27}H_{52}O_4Si_2$ | 1.84 |
| **27** | (3E,12Z)-nonadeca-1,3,12-triene-5,14-diol | Hydrocarbon | 41.66 | 294.48 | $C_{19}H_{34}O_2$ | 0.70 |
| **28** | Stigmasterol | Steroid | 44.98 | 412.70 | $C_{29}H_{48}O$ | 1.23 |

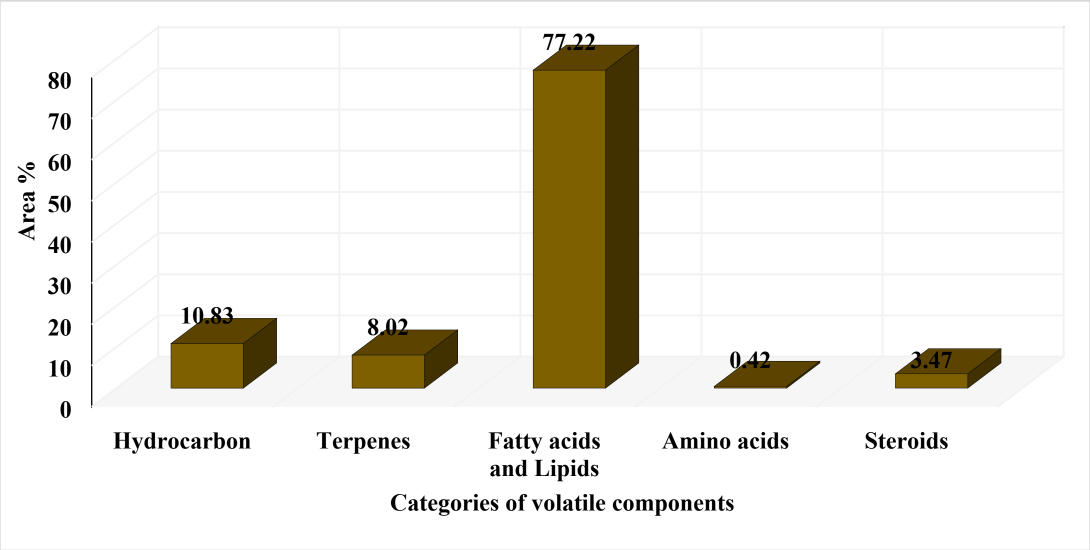


**Figure S1**. The classified chemical categories deduced from *A. articulata* extract by GC-MS mass spectroscopic analysis.

**FTIR Spectroscopy**

**Table S2.** Condensed FTIR spectral assignments of *A. articulata* extract, Ag-$Fe_2O_3$ nanocomposite, and Ag-Co nanocomposite, indicating functional groups and corresponding interpretations.

| Sample(s) | Wavenumber ($cm^{-1}$) | Functional group | Interpretation |
|---|---|---|---|
| ***A. articulata*** **extract & Ag-$Fe_2O_3$ NC** | 3346, 3197 | O-H / N-H stretching | Phenolic compounds, alcohols, and amines from phytochemicals acting as capping agents |
| | 1715 | C=O stretching | Carbonyl groups in aldehydes, ketones, and carboxylic acids |
| | 1600 | C=C stretching | Aromatic rings from polyphenols |
| | 1447, 1421 | C-H bending | Alkanes and aromatic hydrocarbons |
| | 1350, 1307 | C-N stretching | Amines and proteins from plant metabolites |
| | 1183, 1081, 1024 | C-O stretching | Alcohols, ethers, and esters |
| | 998, 915, 809 | =C-H bending | Alkenes and aromatic out-of-plane bending |
| | 664, 623, 596 | Fe-O / Ag-O stretching | Formation of $Fe_2O_3$ and Ag phases |
| | 494, 464, 427 | Lattice vibrations | Crystalline metal oxide phases |
| **Ag-Co NC** | 3338 | O-H / N-H stretching | Phenolics, alcohols, and amines as stabilizers |
| | 1627 | C=C / N-H bending | Aromatic residues and amide groups |
| | 1445 | C-H bending | Hydrocarbon groups |
| | 1387 | C-H deformation | Aliphatic compounds |
| | 1308 | C-N stretching | Amines/proteins |
| | 1037 | C-O stretching | Alcohols and ethers |
| | 819 | =C-H bending | Aromatic out-of-plane vibrations |
| | 591 | Co-O stretching | Formation of cobalt oxide phase |

## Zeta Potential Analysis

**Table S3**. Zeta potential and electrophoretic mobility values of green-synthesized $Ag/Fe_2O_3$ and Ag/Co nanocomposites.

| Nanocomposite | Zeta Potential (mV) | Electrophoretic Mobility ($cm^2/Vs$) |
|---|---|---|
| **$Ag/Fe_2O_3$ NC** | -22.4 | -0.000173 |
| **Ag/Co NC** | -1.1 | -0.000009 |

## HR-TEM

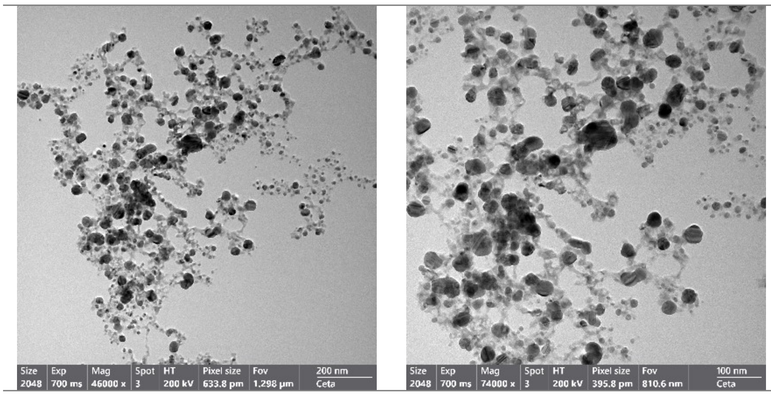

**(a) Ag/Co NC**

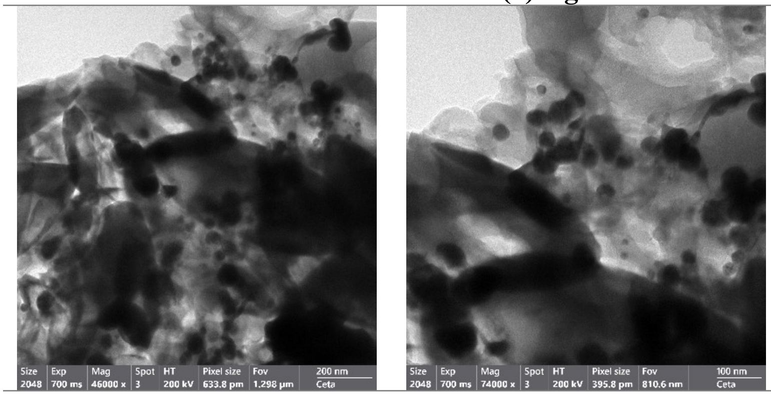

**(b) $Ag/Fe_2O_3$ NC**

**Figure S2**. HR-TEM micrographs of (a) Ag/Co, and (b) $Ag/Fe_2O_3$ NCs.

**SEM**

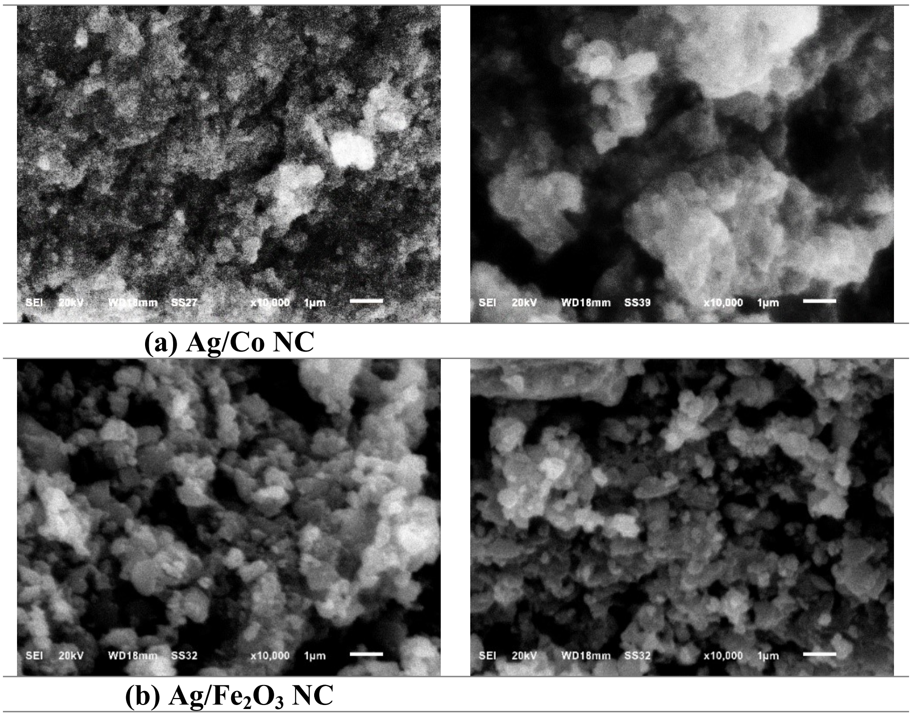


**Figure S3**. SEM micrographs of (a) Ag/Co, and (b) Ag/$Fe_2O_3$ NCs.

**XRD Analysis**

**Table S4. Peak List identified from XRD analysis of Ag/Co NC.**

| Pos. [°2Th.] | Height [cts] | FWHM [°2Th.] | d-spacing [Å] | Rel. Int. [%] | Tip width [°2Th.] |
|---|---|---|---|---|---|
| 27.8987 | 5.31 | 0.2362 | 3.19805 | 10.36 | 0.2834 |
| 32.2830 | 17.36 | 0.1181 | 2.77305 | 33.89 | 0.1417 |
| 38.1470 | 51.23 | 0.3936 | 2.35918 | 100.00 | 0.4723 |
| 44.3937 | 11.81 | 0.7872 | 2.04065 | 23.06 | 0.9446 |
| 46.3297 | 7.64 | 0.2362 | 1.95979 | 14.92 | 0.2834 |
| 64.5517 | 14.47 | 0.6298 | 1.44371 | 28.25 | 0.7557 |
| 77.6346 | 17.02 | 0.4723 | 1.22988 | 33.22 | 0.5668 |
| 81.6952 | 6.29 | 0.5760 | 1.17774 | 12.27 | 0.6912 |

**Table S5. Peak List identified from XRD analysis of $Ag/Fe_2O_3$ NC.**

| Pos. [°2Th.] | Height [cts] | FWHM [°2Th.] | d-spacing [Å] | Rel. Int. [%] | Tip width [°2Th.] |
|---|---|---|---|---|---|
| 14.9574 | 18.58 | 0.1181 | 5.92310 | 30.24 | 0.1417 |
| 22.3139 | 19.12 | 0.1181 | 3.98423 | 31.10 | 0.1417 |
| 24.2788 | 10.99 | 0.2362 | 3.66606 | 17.88 | 0.2834 |
| 28.1323 | 44.95 | 0.1574 | 3.17203 | 73.14 | 0.1889 |
| 29.2071 | 47.76 | 0.1574 | 3.05770 | 77.71 | 0.1889 |
| 31.1366 | 61.46 | 0.1181 | 2.87249 | 100.00 | 0.1417 |
| 32.2928 | 24.54 | 0.1574 | 2.77223 | 39.92 | 0.1889 |
| 33.8900 | 54.84 | 0.1574 | 2.64514 | 89.22 | 0.1889 |
| 35.5398 | 21.93 | 0.1181 | 2.52604 | 35.68 | 0.1417 |
| 37.1719 | 22.53 | 0.1968 | 2.41880 | 36.66 | 0.2362 |
| 38.1863 | 14.65 | 0.1968 | 2.35685 | 23.84 | 0.2362 |
| 40.5397 | 10.34 | 0.3149 | 2.22530 | 16.83 | 0.3779 |
| 44.4047 | 4.98 | 0.6298 | 2.04017 | 8.10 | 0.7557 |
| 45.8380 | 22.14 | 0.1574 | 1.97966 | 36.03 | 0.1889 |
| 47.2018 | 17.17 | 0.2362 | 1.92559 | 27.94 | 0.2834 |
| 49.7261 | 9.28 | 0.2362 | 1.83360 | 15.10 | 0.2834 |
| 53.5497 | 8.29 | 0.2362 | 1.71135 | 13.49 | 0.2834 |
| 54.9223 | 7.29 | 0.2362 | 1.67178 | 11.86 | 0.2834 |
| 63.0612 | 7.42 | 0.3149 | 1.47420 | 12.07 | 0.3779 |
| 74.4473 | 2.15 | 1.1520 | 1.27337 | 3.50 | 1.3824 |

**Phytochemical Analysis**

**Table S6**. The results of the phytochemical analysis of the investigated extracted samples.

| Samples | Phenolic Content [a] | Flavonoid Content [b] | Tannin Content [c] |
|---|---|---|---|
| ***A. articulata* extract** | 134.13±1.73 | 44.84±1.09 | 17.85±0.09 |
| **$Ag/Fe_2O_3$ NC** | 70.522±1.24 | 38.88±1.15 | 12.22±0.07 |
| **Ag/Co NC** | 22.421±1.16 | 32.55±1.03 | 4.257±0.05 |

[a] Phenolic Content "mg gallic acid/1 gm dry sample"

[b] Flavonoid Content "mg catechin acid/1 gm dry sample"

[c] Tannins Contents "mg tannic acid acid/1 gm dry sample"

## Antioxidant Activity

### DPPH Assay

**Table S7**. The Antioxidant Results by DPPH assay.

| Samples | Concentrations (mg/mL) | % Remaining DPPH | % Scavenging Activity | $IC_{50}$ (mg/mL) |
|---|---|---|---|---|
| *A. articulata* extract | 0.165 | 13.62±1.53 | 86.38±1.53 | 0.057±1.23 |
| | 0.083 | 35.95±1.07 | 64.05±1.07 | |
| | 0.041 | 64.49±1.29 | 35.51±1.29 | |
| | 0.021 | 74.95±1.02 | 25.05±1.02 | |
| $Ag/Fe_2O_3$ NC | 0.443 | 15.69±1.33 | 84.31±1.33 | 0.096±1.02 |
| | 0.222 | 32.35±0.08 | 67.65±0.08 | |
| | 0.111 | 47.06±1.21 | 52.94±1.21 | |
| | 0.055 | 58.5±1.47 | 41.5±1.47 | |
| Ag/Co NC | 0.808 | 25.93±1.83 | 74.07±1.83 | 0.271±1.02 |
| | 0.404 | 41.5±1.06 | 58.5±1.06 | |
| | 0.202 | 53.81±1.10 | 46.19±1.10 | |
| | 0.101 | 63.29±0.07 | 36.71±0.07 | |
| Ascorbic acid | 0.06 | 15.27±1.34 | 84.73±1.34 | 0.022±1.40 |
| | 0.03 | 39.08±1.09 | 60.92±1.09 | |
| | 0.02 | 61.07±1.83 | 38.93±1.83 | |
| | 0.01 | 74.81±1.03 | 25.19±1.03 | |

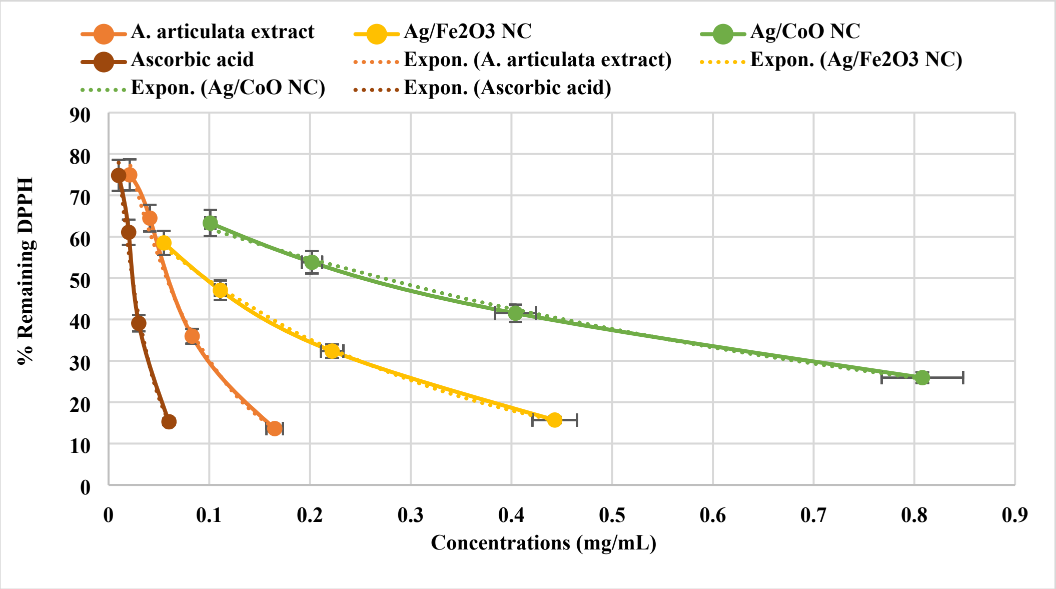


**Figure S4**. The relationship between sample concentration (mg/mL) versus % Remaining DPPH.

**Ferric Reducing Power Assay**

**Table S8**. The results of antioxidant activity by Ferric reducing power assay.

| Sample | Concentration (mg/mL) | Absorbance read |
|---|---|---|
| ***A. articulata* extract** | 10.58 | 1.737±1.27 |
| **Ag/$Fe_2O_3$ NC** | 10.58 | 1.357±1.51 |
| **Ag/Co NC** | 10.58 | 0.855±1.08 |

**Antibacterial Activity**

**Table S9**. The results of antibacterial activity of *A. articulata* extract and nanocomposites.

| **Microorganisms** | **Inhibition zones in mm** | | | |
|---|---|---|---|---|
| ***Gram-negative bacteria*** | ***A. articulata* extract** | **Ag/$Fe_2O_3$ NC** | **Ag/Co NC** | **Azithromycin** |
| *Escherichia coli* (ATCC 10536) | NA | 22.0 ± 1.38 | 13.0 ± 16.3 | 27.0 ± 1.46 |
| *Salmonella typhimurium*(ATCC 25566) | NA | 19.0 ± 1.52 | 13.0 ± 1.19 | NA |
| *Klebsiella pneumonia* (ATCC 10031) | NA | 23.0 ± 2.06 | 14.0 ± 1.80 | 20.0 ± 1.62 |
| *Enterobacter cloacae* (DMS 30054) | NA | 21.0 ± 1.82 | 15.0 ± 1.64 | 13.0 ± 1.17 |
| ***Gram-positive bacteria*** | | | | |
| *Bacillus subtilis* (DMS 1088) | NA | 24.0 ± 1.83 | NA | 22.0 ± 1.90 |
| *Bacillus cereus* (EMCC number 1080) | NA | 24.0 ± 1.61 | 13.0 ± 1.29 | 15.0 ± 1.06 |
| *Staphylococcus aureus* (ATCC 6538) | NA | 24.0 ± 1.72 | 14.0 ± 1.07 | 24.0 ± 1.55 |
| *Staphylococcus epidermidis* (EMCC number 1353ᵗ) | NA | 24.0 ± 1.40 | 14.0 ± 1.39 | 21.0 ± 1.19 |

NA: refers to no activity. The results are expressed as the mean value ± standard deviation (SD). The experiments were run for triplicate. **NA**: no activity; **ns**: not significant; results expressed as mean ± SD (triplicate). The statistical significance vs Azithromycin indicated $*p < 0.05$ (Ag/$Fe_2O_3$ NC), and ns (Ag/Co NC).

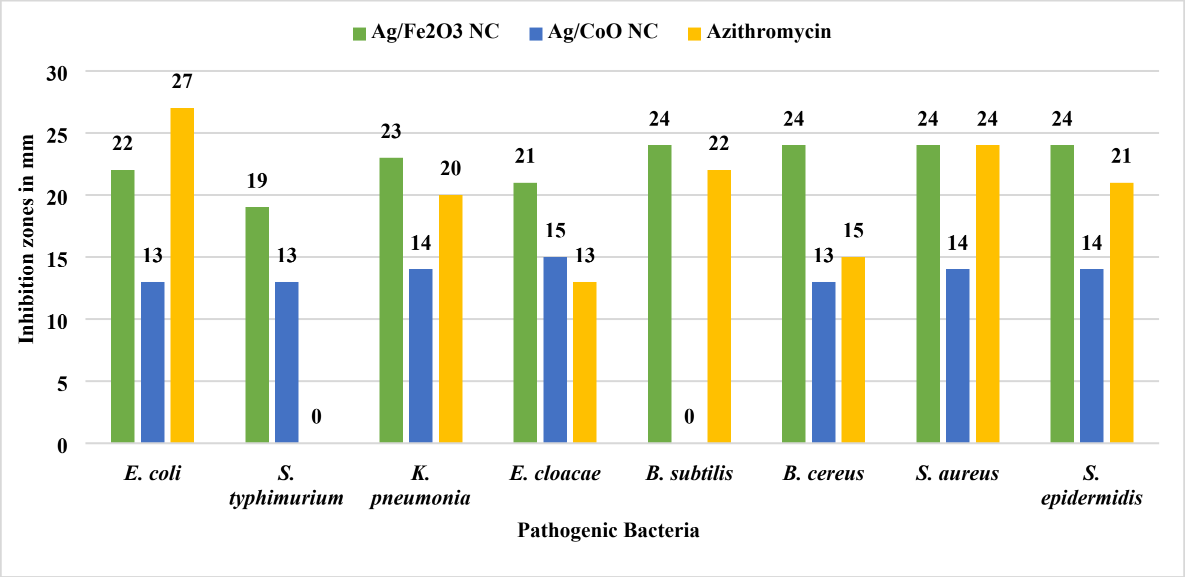


**Figure S5**. Antibacterial activity of Ag/$Fe_2O_3$ and Ag/Co nanocomposites in comparison with Azithromycin against pathogenic bacteria.

**Minimum Inhibitory Concentration (MIC)**

***1. K. pneumonia***

**Table S10**. MIC results of Ag/$Fe_2O_3$ NC against *K. pneumonia.*

| Test tube no. | **Concentration (mg/mL)** | **O.D$_{600}$** |
|---|---|---|
| 1 | 7.09 | 0.001 |
| 2 | 3.545 | 0.018 |
| **3** | 1.7725 | **0.024** |
| 4 | 0.8863 | 1.143 |
| 5 | 0.4431 | 1.637 |
| 6 | 0.2216 | 1.752 |
| 7 | 0.1108 | 1.881 |
| 8 | 0.0554 | 1.943 |

After 24 h of incubation at 37 °C, turbidity was observed in test tube 4, whereas in tube 3 (MIC), no turbidity was seen, exhibiting inhibition of bacterial growth.

**Table S11**. MIC results of Ag/Co NC against *K. pneumonia.*

| Test tube no. | **Concentration (mg/mL)** | **O.D$_{600}$** |
|---|---|---|
| 1 | 7.09 | 0.001 |
| 2 | 3.545 | 0.009 |
| 3 | 1.7725 | 0.011 |
| 4 | 0.8863 | 0.012 |
| 5 | 0.4431 | 0.016 |
| 6 | 0.2216 | **0.053** |
| 7 | 0.1108 | 0.348 |
| 8 | 0.0554 | 0.462 |

After 24 h of incubation at 37 °C, turbidity was noticed in test tube 7, whereas in tube 6 (MIC), no turbidity was perceived, exhibiting inhibition of bacterial growth.

***2. S. aureus***

**Table S12**. MIC results of Ag/$Fe_2O_3$ NC NC against *S. aureus.*

| Test tube no. | **Concentration (mg/mL)** | **$O.D_{600}$** |
|---|---|---|
| 1 | 7.09 | 0.003 |
| 2 | 3.545 | 0.008 |
| 3 | 1.7725 | **0.051** |
| 4 | 0.8863 | 0.449 |
| 5 | 0.4431 | 0.514 |
| 6 | 0.2216 | 0.670 |
| 7 | 0.1108 | 0.758 |
| 8 | 0.0554 | 0.883 |

After 24 h of incubation at 37 °C, turbidity was noticed in test tube 4, whereas in tube 3 (MIC), no turbidity was seen, exhibiting inhibition of bacterial growth.

**Table S13**. MIC results of Ag/Co NC against *S. aureus.*

| Test tube no. | **Concentration (mg/mL)** | **$O.D_{600}$** |
|---|---|---|
| 1 | 7.09 | 0.011 |
| 2 | 3.545 | **0.014** |
| 3 | 1.7725 | 0.352 |
| 4 | 0.8863 | 0.479 |
| 5 | 0.4431 | 0.519 |
| 6 | 0.2216 | 0.664 |
| 7 | 0.1108 | 0.801 |
| 8 | 0.0554 | 1.019 |

After 24 h of incubation at 37 °C, turbidity was noticed in test tube 3, whereas in tube 2 (MIC), no turbidity was seen, exhibiting inhibition of bacterial growth.

## Insecticidal Activity

**Table S14.** Toxicity of *A. articulata* extract and nanocomposites on *Aphis craccivora* after one day under laboratory conditions.

| Treatments | Conc (ppm) | Insecticidal activity after 24 hours of treatment | | | | | | | | | |
|---|---|---|---|---|---|---|---|---|---|---|---|
| | | Mortality % | $LC_{50}$ (ppm) | Confidence limit (ppm) | | $LC_{90}$ (ppm) | Confidence limit (ppm) | | *Slope* ± *S.E.* | *R* | Toxicity Index (%) |
| | | | | Lower | Upper | | Lower | Upper | | | |
| *A. articulata* | 50 | 27.37±0.98$^{E}$ | 167.63 | 162.25 | 173.01 | 389.24 | 383.86 | 394.62 | 2.51±0.29 | 0.9911 | 23.11 |
| | 100 | 37.89±1.06$^{D}$ | | | | | | | | | |
| | 150 | 48.42±1.54$^{C}$ | | | | | | | | | |
| | 200 | 57.95±2.11$^{B}$ | | | | | | | | | |
| | 250 | 62.47±1.98$^{A}$ | | | | | | | | | |
| | $LSD_{0.05}$ | 4.44 | | | | | | | | | |
| | *P value* | < 0.0001*** | | | | | | | | | |
| Ag/Co NC | 5 | 15.32±0.40$^{E}$ | 34.52 | 23.29 | 45.75 | 89.93 | 78.70 | 101.16 | 3.04±0.40 | 0.9325 | 112.19 |
| | 10 | 31.84±0.87$^{D}$ | | | | | | | | | |
| | 25 | 52.63±1.48$^{C}$ | | | | | | | | | |
| | 50 | 73.16±2.71$^{B}$ | | | | | | | | | |
| | 100 | 89.62±2.88$^{A}$ | | | | | | | | | |
| | $LSD_{0.05}$ | 3.98 | | | | | | | | | |
| | *P value* | < 0.0001*** | | | | | | | | | |
| $Ag/Fe_2O_3$ NC | 5 | 11.05±0.32$^{E}$ | 43.83 | 32.91 | 54.75 | 100.21 | 89.29 | 111.13 | 2.83±0.35 | 0.9413 | 88.36 |
| | 10 | 26.58±0.55$^{D}$ | | | | | | | | | |
| | 25 | 41.37±1.48$^{C}$ | | | | | | | | | |
| | 50 | 67.89±1.96$^{B}$ | | | | | | | | | |
| | 100 | 82.42±2.34$^{A}$ | | | | | | | | | |
| | $LSD_{0.05}$ | 3.64 | | | | | | | | | |
| | *P value* | < 0.0001*** | | | | | | | | | |
| Azadirachtin (Okios 3.2% EC) | 5 | 15.79±0.20$^{E}$ | 38.46 | 27.48 | 49.98 | 91.47 | 81.22 | 103.72 | 2.74±0.30 | 0.9625 | 100 |
| | 10 | 26.32±0.61$^{D}$ | | | | | | | | | |
| | 25 | 47.37±0.84$^{C}$ | | | | | | | | | |
| | 50 | 68.42±1.15$^{B}$ | | | | | | | | | |
| | 100 | 90.37±2.05$^{A}$ | | | | | | | | | |
| | $LSD_{0.05}$ | 4.11 | | | | | | | | | |
| | *P value* | < 0.0001*** | | | | | | | | | |

% Mortality was expressed as mean ± SE (standard error) of 3 replicates. $LC_{50}$; $LC_{90}$; upper limit; lower limit their confidence limits at 95%. Different letter per each treatment mean values significance at probability level of 0.05. *** p < 0.001.

## Insecticidal Activity

**Table S15.** Toxicity of *A. articulata* extract and nanocomposites on *Brevicoryne brassicae* after one day under laboratory conditions.

| Treatments | Conc (ppm) | Insecticidal activity after 24 hours of treatment | | | | | | | | | |
|---|---|---|---|---|---|---|---|---|---|---|---|
| | | Mortality % | $LC_{50}$ (ppm) | Confidence limit (ppm) | | $LC_{90}$ (ppm) | Confidence limit (ppm) | | *Slope* ± *S.E.* | R | Toxicity Index (%) |
| | | | | Lower | Upper | | Lower | Upper | | | |
| | 50 | 22.68±0.61$^{E}$ | | | | | | | | | |
| | 100 | 40.37±1.09$^{D}$ | | | | | | | | | |
| | 150 | 52.67±1.42$^{C}$ | | | | | | | | | |
| *A. articulata* | 200 | 60.82±1.64$^{B}$ | 149.96 | 142.99 | 156.93 | 313.69 | 306.72 | 320.66 | 0.257±3.561 | 0.9918 | 18.23 |
| | 250 | 73.54±2.01$^{A}$ | | | | | | | | | |
| | $LSD_{0.05}$ | 5.33 | | | | | | | | | |
| | *P value* | < 0.0001*** | | | | | | | | | |
| | 5 | 17.93±0.48$^{E}$ | | | | | | | | | |
| | 10 | 35.42±0.96$^{D}$ | | | | | | | | | |
| | 25 | 59.21±1.60$^{C}$ | | | | | | | | | |
| Ag/Co NC | 50 | 81.74±2.21$^{B}$ | 26.93 | 15.01 | 38.85 | 76.90 | 64.98 | 88.82 | 0.356±6.083 | 0.9330 | 101.35 |
| | 100 | 100±2.70$^{A}$ | | | | | | | | | |
| | $LSD_{0.05}$ | 4.91 | | | | | | | | | |
| | *P value* | < 0.0001*** | | | | | | | | | |
| | 5 | 14.09±0.38$^{E}$ | | | | | | | | | |
| | 10 | 28.92±0.78$^{D}$ | | | | | | | | | |
| | 25 | 43.41±1.17$^{C}$ | | | | | | | | | |
| $Ag/Fe_2O_3$ NC | 50 | 70.93±1.92$^{B}$ | 32.27 | 20.81 | 43.73 | 75.12 | 63.66 | 86.58 | 0.287±5.848 | 0.9662 | 84.63 |
| | 100 | 93.46±2.53$^{A}$ | | | | | | | | | |
| | $LSD_{0.05}$ | 3.95 | | | | | | | | | |
| | *P value* | < 0.0001*** | | | | | | | | | |
| | 5 | 21.35±0.58$^{E}$ | | | | | | | | | |
| | 10 | 31.88±0.86$^{D}$ | | | | | | | | | |
| | 25 | 52.93±1.43$^{C}$ | | | | | | | | | |
| Azadirachtin (Okios 3.2% EC) | 50 | 87.98±2.38$^{B}$ | 27.29 | 15.01 | 39.57 | 75.82 | 63.54 | 88.10 | 0.347±6.266 | 0.9326 | 100 |
| | 100 | 100±2.70$^{A}$ | | | | | | | | | |
| | $LSD_{0.05}$ | 3.89 | | | | | | | | | |
| | *P value* | < 0.0001*** | | | | | | | | | |

Mortality was expressed as mean ± SE (standard error) of 3 replicates. $LC_{50}$; $LC_{90}$; upper limit; lower limit their confidence limits at 95%. Different letter per each treatment mean values significance at probability level of 0.05. *** $p < 0.001$.

## Reference


[1] I. de Dobbeleer, J. Gummersbach, H.-J. Huebschmann, A. Mayer and P. Silcock, Thermo Scientific TSQ Quantum XLS Ultra GC-MS/MS in EI-SRM mode, Thermo Fisher Scientific, Dreieich, Germany, 2012, 1-6.
